# Supplementary figures and images for: Predicting climate-change induced heat-related illness risk in Grand Canyon National Park visitors
Source: PLoS One. 2023 Aug 9;18(8):e0288812. doi: 10.1371/journal.pone.0288812 (PMC10411749; doi:10.1371/journal.pone.0288812)

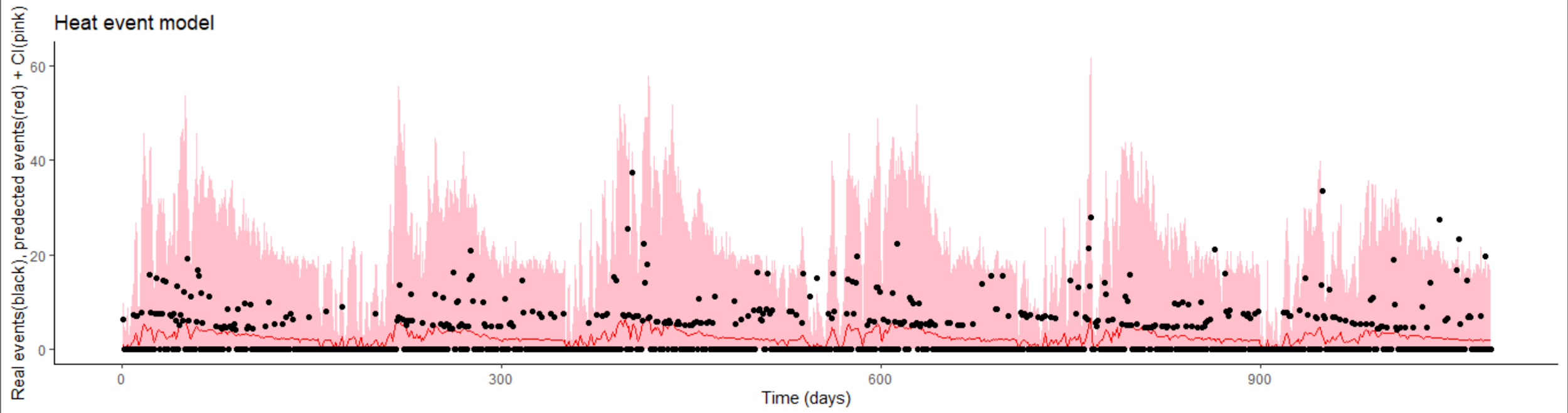

Supplement: S1 Fig — Figure of recorded heat events (black) and predicted heat events (red line) at Grand Canyon National Park from 2004–2009; the pink shaded areas representing 95% prediction confidence intervals from the final negative binomial regression model. (TIF) [file pone.0288812.s001.tif]
