# Supplementary material for: Predicting climate-change induced heat-related illness risk in Grand Canyon National Park visitors
Source: PLoS One. 2023 Aug 9;18(8):e0288812. doi: 10.1371/journal.pone.0288812 (PMC10411749; doi:10.1371/journal.pone.0288812)
Supplement: S4 File — Rmd file with code and output for regression model fitting. (HTML) [file pone.0288812.s005.html]

Heat code model fit


# Heat code model fit

#### Brinkley Raynor

#### 2021

# Load data

```
setwd("~/GRCA_HeatProject")
dfC <- read.csv("HeatData_Count.csv") %>% na.omit(HeatDiff) #individual days
dfB <- read.csv("HeatData_Week.csv")#binned by week
```

# Day level data (not binned)

## Build model

Method: Hosmer, Lemeshow and Sturdivant: Applied Logistic Regression (2013), Ch4

1. Construct an initial main-effects model using explanatory variables ajd others that show evidence of being relevant when used as sole predictors

```
######################################################################################################
#Check for sole predictors
######################################################################################################
#Null
nb.null <- glm(Events~ 1 +offset(log(Visitors)),
              family=negative.binomial(theta =1), data=dfC)
#summary(nb.null)

#Heat index
nb.HI <- glm(Events~ Heat_Index +offset(log(Visitors)),
              family=negative.binomial(theta =1), data=dfC)
#summary(nb.HI) #p=1.31e-10 


#month (factor)
nb.month <- glm(Events~ factor(month) +offset(log(Visitors)),
              family=negative.binomial(theta =1), data=dfC)
#summary(nb.month) #For factor, need to test joint significance:

#weekday
nb.weekday <- glm(Events~weekday +offset(log(Visitors)),
              family=negative.binomial(theta =1), data=dfC)
#summary(nb.weekday) #For factor, need to test joint significance:

#day(of the year)
nb.day <- glm(Events~ day +offset(log(Visitors)),
              family=negative.binomial(theta =1), data=dfC)
#summary(nb.day) #p= 0.51  ---> leave out of initial model

#difference in Heat Index from previous day
nb.diff <- glm(Events~ HeatDiff +offset(log(Visitors)),
              family=negative.binomial(theta =1), data=dfC)
#summary(nb.diff) #p=0.0186

#difference in heat ratio from previous day
nb.ratio <- glm(Events~ HeatRatio +offset(log(Visitors)),
              family=negative.binomial(theta =1), data=dfC)
#summary(nb.ratio) #p=0.0259   #plan on including just diff to avoid colinearities


######################################################################################################
#Initial main effects model
######################################################################################################
nb.init<- glm(Events~ Heat_Index + factor(month) + HeatDiff +offset(log(Visitors)),
              family=negative.binomial(theta =1), data=dfC)
#summary(nb.init)

#anova(nb.null, nb.init, test="LR") #p= 2.69e-11
```

**Take away:** Heat index, month and difference from previous day were all significant and incorporated into an initial main effects model. The initial model does significantly better than the null by LR test.

2. Conduct backward elimination, keeping a cariable if it is either a somewhat more stringent level or shows evidence of being a relevant confounder, in the sense that the estimated effect of a key variable changes substantially when removed

```
nb.omit_HI <- glm(Events~ factor(month) + HeatDiff +offset(log(Visitors)),
              family=negative.binomial(theta =1), data=dfC)
#summary(nb.omit_HI)
#anova(nb.omit_HI, nb.init, test="LR") #p-value =8.927e-12 --> keep HI

nb.omit_month <- glm(Events~ Heat_Index + HeatDiff +offset(log(Visitors)),
              family=negative.binomial(theta =1), data=dfC)
#summary(nb.omit_month)
#anova(nb.omit_month, nb.init, test="LR") #p-value =6.296e-09---> keep month

nb.omit_diff<- glm(Events~ Heat_Index + factor(month) +offset(log(Visitors)),
              family=negative.binomial(theta =1), data=dfC)
#summary(nb.omit_diff)
#anova(nb.omit_diff, nb.init, test="LR") #p-value =0.9962
#Interpretation: Model without difference in day as a variable is not significantly different than model with it.
#Estimated effects of other key variables do not change significantly
#Check diff ratio to see if same thing

nb.init2<- glm(Events~ Heat_Index + factor(month) + HeatRatio +offset(log(Visitors)),
              family=negative.binomial(theta =1), data=dfC)
#summary(nb.init2)
#anova(nb.omit_diff, nb.init2, test="LR") #0.1936
#still not significant
```

**Take away:** eliminated difference in HI between days, bc no significant improvement than in model without

3. Add to the model any variables that were not included in step 1 but that are significant when adjusting for the variables in the model after step 2, since a variable may not be significantly associated with y but may make an important contribution to the presence of other variables

```
#day of year
nb.add_days <- glm(Events~ Heat_Index + factor(month) +day+offset(log(Visitors)),
              family=negative.binomial(theta =1), data=dfC)
              
#summary(nb.add_days)
#anova(nb.omit_diff, nb.add_days, test="LR") #p = 0.7071 --> no significant improvement

#day of week
nb.add_weekday <- glm(Events~ Heat_Index + factor(month) +weekday+offset(log(Visitors)),
              family=negative.binomial(theta =1), data=dfC)
              
#summary(nb.add_weekday)
#anova(nb.omit_diff, nb.add_weekday, test="LR") #p = 3824 --> no significant improvement
```

**take away: no significant improvement in model when adding back in previously insignificant adjusters**

4. Check for plausible interactions among variables in the model using significace tests at conventional levels such as 0.05

```
nb.int <- glm(Events~ Heat_Index + factor(month) + Heat_Index*factor(month) +offset(log(Visitors)),
              family=negative.binomial(theta =1), data=dfC)
#summary(nb.int)
#anova(nb.omit_diff, nb.int, test="LR") #p = 0.3167 1--> no significant difference

#going to check interactions with diff just in case
nb.int2 <- glm(Events~ Heat_Index + factor(month) + HeatDiff*factor(month) +offset(log(Visitors)),
              family=negative.binomial(theta =1), data=dfC)
#anova(nb.omit_diff, nb.int2, test="LR") #p=0.7582 --> no sign difference

nb.int3 <- glm(Events~ Heat_Index + factor(month) + HeatDiff*Heat_Index +offset(log(Visitors)),
              family=negative.binomial(theta =1), data=dfC)
#summary(nb.int3)
#anova(nb.omit_diff, nb.int3, test="LR") #p=0.7309
```

**Take away:** no significant improvement in model when looking at interactions

5. Conduct follow-up diagnostics

```
#Going to examine single variable (month, HI, diff) alone, the initial model (month + HI +diff), the reduced model (month + HI) and the interaction model (month + HI + diff*HI)
df.stat <- data.frame("Model" = c("nb.init", "nb.HI", "nb.month", 
                                   "nb.diff", "nb.omit_diff", "nb.int3", "nb.null"),
                      "AIC" = c(nb.init$aic, nb.HI$aic, nb.month$aic, nb.diff$aic, nb.omit_diff$aic, nb.int3$aic, nb.null$aic),
                      "Deviance" = c(nb.init$deviance, nb.HI$deviance, nb.month$deviance, nb.diff$deviance, nb.omit_diff$deviance, nb.int3$deviance, nb.null$deviance),
                      "CV"= c(boot::cv.glm(dfC, nb.init, K = 10)$delta[[2]],
                              boot::cv.glm(dfC, nb.HI, K = 10)$delta[[2]],
                              boot::cv.glm(dfC, nb.month, K = 10)$delta[[2]],
                              boot::cv.glm(dfC, nb.diff, K = 10)$delta[[2]],
                              boot::cv.glm(dfC, nb.omit_diff, K = 10)$delta[[2]],
                              boot::cv.glm(dfC, nb.int3, K = 10)$delta[[2]],
                              boot::cv.glm(dfC, nb.null, K = 10)$delta[[2]]))
```

**Take away: this confirms what we found with forward selection w LRT- the nb.omit\_diff model (Events~ Heat\_Index + factor(month) +offset(log(Visitors))) does the best by lowest AIC and lowest CV prediction error**

## Cross validate

70:30 split random

```
#check
set.seed(2357)
dfC$train <- sample(100, size = nrow(dfC), replace = TRUE)
dfC$train <- ifelse(dfC$train > 70, 0, 1)

#train prediction
nb.null_train <- glm(Events~ 1 +offset(log(Visitors)),
              family=negative.binomial(theta =1), data=dfC%>%filter(train==1))

nb.HI_train <- glm(Events~ Heat_Index +offset(log(Visitors)),
              family=negative.binomial(theta =1), data=dfC%>%filter(train==1))

nb.month_train <- glm(Events~ factor(month) +offset(log(Visitors)),
              family=negative.binomial(theta =1), data=dfC%>%filter(train==1))

nb.weekday_train <- glm(Events~weekday +offset(log(Visitors)),
              family=negative.binomial(theta =1), data=dfC%>%filter(train==1))

nb.day_train <- glm(Events~ day +offset(log(Visitors)),
              family=negative.binomial(theta =1), data=dfC%>%filter(train==1))

nb.diff_train <- glm(Events~ HeatDiff +offset(log(Visitors)),
              family=negative.binomial(theta =1), data=dfC%>%filter(train==1))

nb.ratio_train <- glm(Events~ HeatRatio +offset(log(Visitors)),
              family=negative.binomial(theta =1), data=dfC%>%filter(train==1))

nb.omit_diff_train<- glm(Events~ Heat_Index + factor(month) +offset(log(Visitors)),
              family=negative.binomial(theta =1), data=dfC%>%filter(train==1))

nb.HI_train <- glm(Events~ Heat_Index +offset(log(Visitors)),
              family=negative.binomial(theta =1), data=dfC)

nb.month_train <- glm(Events~ factor(month) +offset(log(Visitors)),
              family=negative.binomial(theta =1), data=dfC%>%filter(train==1))

nb.weekday_train <- glm(Events~weekday +offset(log(Visitors)),
              family=negative.binomial(theta =1), data=dfC%>%filter(train==1))

nb.init_train<- glm(Events~ Heat_Index + factor(month) + HeatDiff +offset(log(Visitors)),
              family=negative.binomial(theta =1), data=dfC%>%filter(train==1))

nb.day_train <- glm(Events~ day +offset(log(Visitors)),
              family=negative.binomial(theta =1), data=dfC%>%filter(train==1))

nb.diff_train <- glm(Events~ HeatDiff +offset(log(Visitors)),
              family=negative.binomial(theta =1), data=dfC%>%filter(train==1))

nb.ratio_train <- glm(Events~ HeatRatio +offset(log(Visitors)),
              family=negative.binomial(theta =1), data=dfC%>%filter(train==1))

nb.int3_train <- glm(Events~ Heat_Index + factor(month) + HeatDiff*Heat_Index +offset(log(Visitors)),
              family=negative.binomial(theta =1), data=dfC%>%filter(train==1))


#apply prediction
pred.train <- dfC%>%filter(train==0) %>%
  mutate(init      = predict(nb.init_train, newdata=dfC%>%filter(train==0),type='response'),
         HI        = predict(nb.HI_train, newdata=dfC%>%filter(train==0),type='response'),
         month     = predict(nb.month_train, newdata=dfC%>%filter(train==0),type='response'),
         diff      = predict(nb.diff_train, newdata=dfC%>%filter(train==0),type='response'),
         omit_diff = predict(nb.omit_diff_train, newdata=dfC%>%filter(train==0),type='response'),
         int3      = predict(nb.int3_train, newdata=dfC%>%filter(train==0),type='response'),
         null      = predict(nb.null_train, newdata=dfC%>%filter(train==0),type='response'))


df.stat$train.MSE <- c(MLmetrics::MSE(pred.train$Events, pred.train$init),
                 MLmetrics::MSE(pred.train$Events, pred.train$HI),
                 MLmetrics::MSE(pred.train$Events, pred.train$month),
                 MLmetrics::MSE(pred.train$Events, pred.train$diff),
                 MLmetrics::MSE(pred.train$Events, pred.train$omit_diff),
                 MLmetrics::MSE(pred.train$Events, pred.train$int3),
                 MLmetrics::MSE(pred.train$Events, pred.train$null))

#apply regressions to test data
pred.test <- dfC%>%filter(train==1) %>%
  mutate(init      = predict(nb.init_train, newdata=dfC%>%filter(train==1),type='response'),
         HI        = predict(nb.HI_train, newdata=dfC%>%filter(train==1),type='response'),
         month     = predict(nb.month_train, newdata=dfC%>%filter(train==1),type='response'),
         diff      = predict(nb.diff_train, newdata=dfC%>%filter(train==1),type='response'),
         omit_diff = predict(nb.omit_diff_train, newdata=dfC%>%filter(train==1),type='response'),
         int3      = predict(nb.int3_train, newdata=dfC%>%filter(train==1),type='response'),
         null      = predict(nb.null_train, newdata=dfC%>%filter(train==1),type='response'))

df.stat$test.mse <-  c(MLmetrics::MSE(pred.test$Events, pred.test$init),
                       MLmetrics::MSE(pred.test$Events, pred.test$HI),
                       MLmetrics::MSE(pred.test$Events, pred.test$month),
                       MLmetrics::MSE(pred.test$Events, pred.test$diff),
                       MLmetrics::MSE(pred.test$Events, pred.test$omit_diff),
                       MLmetrics::MSE(pred.test$Events, pred.test$int3),
                       MLmetrics::MSE(pred.test$Events, pred.test$null))

df.stat
```

```
##          Model      AIC Deviance        CV train.MSE  test.mse
## 1      nb.init 1856.825 746.1626 0.5342002 0.6979591 0.4457215
## 2        nb.HI 1885.087 786.4251 0.5564434 0.7179946 0.4790671
## 3     nb.month 1895.668 789.0057 0.5630724 0.7257182 0.4797710
## 4      nb.diff 1920.591 821.9291 0.5782468 0.7439680 0.4999048
## 5 nb.omit_diff 1854.825 746.1626 0.5332212 0.6979398 0.4457105
## 6      nb.int3 1858.298 745.6356 0.5332440 0.6970941 0.4456910
## 7      nb.null 1923.340 826.6779 0.5795748 0.7427481 0.5011393
```

# Binned data (by week)

Instead of looking at zero inflated poisson, I binned the data, looking at week statistics. Week is currently defined as CDC epi weeks (Sunay-Satarday)

## Test if data are overdispersed

```
glm.pois <- glm(Events.sum~ 1, data = dfB, family = poisson)
AER::dispersiontest(glm.pois,trafo=1)
```

```
## 
##  Overdispersion test
## 
## data:  glm.pois
## z = 4.3606, p-value = 6.485e-06
## alternative hypothesis: true alpha is greater than 0
## sample estimates:
##    alpha 
## 1.055784
```

**Null hypothesis=no over dispersion —> fail to reject. Because equidispersion, will check poisson as well**

## Build model using HI

Using same forward elimination nethod as above

```
#######################################################################################################
#create negative binomial models
#######################################################################################################
#create nb models
nb.bin.null <- glm.nb(Events.sum ~ 1 + offset(log(visitors.sum)), data = dfB)

#single variables
nb.bin.wk <-glm.nb(Events.sum ~ week + offset(log(visitors.sum)), data = dfB) #not sign
nb.bin.yr <- glm.nb(Events.sum ~ as.factor(year)+ offset(log(visitors.sum)), data = dfB)#not sign
nb.bin.month <- glm.nb(Events.sum ~ as.factor(month)+ offset(log(visitors.sum)), data = dfB) #SIGNIFICANT
nb.bin.Tmax <- glm.nb(Events.sum ~ Tmax.avg+ offset(log(visitors.sum)), data = dfB) #SIGNIFICANT
nb.bin.RH <- glm.nb(Events.sum ~ RHmin.avg+ offset(log(visitors.sum)), data = dfB)#SIGNIFICANT
nb.bin.HI <- glm.nb(Events.sum ~ HI.avg+ offset(log(visitors.sum)), data = dfB)#SIGNIFICANT

#forward selection
nb.bin.init <- glm.nb(Events.sum ~ HI.avg + as.factor(month) +  offset(log(visitors.sum)), data = dfB)
#anova(nb.bin.init, nb.bin.null, test="LR") #p=6.264822e-11
#anova(nb.bin.init, nb.bin.HI, test="LR") #p=4.110749e-08
#anova(nb.bin.init, nb.bin.month, test="LR") #p=7.485405e-07

#check for interactions
nb.bin.int <- glm.nb(Events.sum ~ HI.avg + as.factor(month) + HI.avg*as.factor(month)+ offset(log(visitors.sum)), data = dfB) 
#anova(nb.bin.int, nb.bin.init, test="LR") #p=0.1786232 --> no better

#######################################################################################################
#create poisson models
#######################################################################################################
pois.bin.null <- glm(Events.sum ~ 1 + offset(log(visitors.sum)), family = poisson, data = dfB)

#single variables
pois.bin.wk <-glm(Events.sum ~ week + offset(log(visitors.sum)), family = poisson, data = dfB) #not sign
pois.bin.yr <- glm(Events.sum ~ as.factor(year)+ offset(log(visitors.sum)), family = poisson,data = dfB)#not sign
pois.bin.month <- glm(Events.sum ~ as.factor(month)+ offset(log(visitors.sum)), family = poisson,data = dfB) #SIGNIFICANT
pois.bin.Tmax <- glm(Events.sum ~ Tmax.avg+ offset(log(visitors.sum)), family = poisson,data = dfB) #SIGNIFICANT
pois.bin.RH <- glm(Events.sum ~ RHmin.avg+ offset(log(visitors.sum)), family = poisson, data = dfB)#SIGNIFICANT
pois.bin.HI <- glm(Events.sum ~ HI.avg+ offset(log(visitors.sum)), family = poisson, data = dfB)#SIGNIFICANT

#forward selection
pois.bin.init <- glm(Events.sum ~ HI.avg + as.factor(month) +  offset(log(visitors.sum)), family = poisson, data = dfB)
# anova(pois.bin.init, pois.bin.null, test="LR") #p=< 2.2e-16
# anova(pois.bin.init, pois.bin.HI, test="LR") #p=8.978e-12 
# anova(pois.bin.init, pois.bin.month, test="LR") #p=9.239e-09
#Keep initial main effects model

#check for interactions
pois.bin.int <- glm(Events.sum ~ HI.avg + as.factor(month) + HI.avg*as.factor(month)+ offset(log(visitors.sum)), family= poisson, data = dfB) 
#anova(pois.bin.int, pois.bin.init, test="LR") #p=0.13 --> no better

#######################################################################################################
#Format stats table
#######################################################################################################
df.bin <- data.frame("Model" = c("nb.bin.null", "nb.bin.wk", "nb.bin.yr", 
                                 "nb.bin.month", "nb.bin.Tmax", "nb.bin.RH", 
                                 "nb.bin.HI", "nb.bin.init", "nb.bin.int",
                                 "pois.bin.null", "pois.bin.wk", "pois.bin.yr", 
                                 "pois.bin.month", "pois.bin.Tmax", "pois.bin.RH", 
                                 "pois.bin.HI", "pois.bin.init", "pois.bin.int"),
                      "AIC" = c(nb.bin.null$aic, nb.bin.wk$aic, nb.bin.yr$aic, 
                                nb.bin.month$aic, nb.bin.Tmax$aic, nb.bin.RH$aic, 
                                nb.bin.HI$aic, nb.bin.init$aic, nb.bin.int$aic,
                                pois.bin.null$aic, pois.bin.wk$aic, pois.bin.yr$aic, 
                                pois.bin.month$aic, pois.bin.Tmax$aic, pois.bin.RH$aic, 
                                pois.bin.HI$aic, pois.bin.init$aic, pois.bin.int$aic),
                      "Deviance" = c(nb.bin.null$deviance, nb.bin.wk$deviance, nb.bin.yr$deviance, 
                                     nb.bin.month$deviance, nb.bin.Tmax$deviance, nb.bin.RH$deviance, 
                                     nb.bin.HI$deviance, nb.bin.init$deviance, nb.bin.int$deviance,
                                     pois.bin.null$deviance, pois.bin.wk$deviance, pois.bin.yr$deviance, 
                                     pois.bin.month$deviance, pois.bin.Tmax$deviance, pois.bin.RH$deviance, 
                                     pois.bin.HI$deviance, pois.bin.init$deviance, pois.bin.int$deviance),
                      "CV"= c(boot::cv.glm(dfB, pois.bin.null, K = 10)$delta[[2]],
                              boot::cv.glm(dfB, pois.bin.wk, K = 10)$delta[[2]],
                              boot::cv.glm(dfB, pois.bin.yr, K = 10)$delta[[2]],
                              boot::cv.glm(dfB, pois.bin.month, K = 10)$delta[[2]],
                              boot::cv.glm(dfB, pois.bin.Tmax, K = 10)$delta[[2]],
                              boot::cv.glm(dfB, pois.bin.RH, K = 10)$delta[[2]],
                              boot::cv.glm(dfB, pois.bin.HI, K = 10)$delta[[2]],
                              boot::cv.glm(dfB, pois.bin.init, K = 10)$delta[[2]],
                              boot::cv.glm(dfB, pois.bin.int, K = 10)$delta[[2]]))
```

**Take away: the best model with the binned data is“Events.sum ~ HI.avg + as.factor(month) + offset(log(visitors.sum))” for both poisson and nb models, they are about equivalent in AIC and CV error, poisson does slightly better bc simpler. Also no evidence of overdispersion (above) so would recommend poisson**

## Forward elimination using Tmax + RHmin

Using same method as above. HI, Tmax and RHmin are all significant as single predictors. However, worried about autocorrelation as HI is derived from temp and rel humidity. SO looking at seperate model sets, one considering HI, one considering RH/temp.

```
#######################################################################################################
#create negative binomial models
#######################################################################################################
#create nb models
nb.bin.null <- glm.nb(Events.sum ~ 1 + offset(log(visitors.sum)), data = dfB)

#single variables
nb.bin.wk <-glm.nb(Events.sum ~ week + offset(log(visitors.sum)), data = dfB) #not sign
nb.bin.yr <- glm.nb(Events.sum ~ as.factor(year)+ offset(log(visitors.sum)), data = dfB)#not sign
nb.bin.month <- glm.nb(Events.sum ~ as.factor(month)+ offset(log(visitors.sum)), data = dfB) #SIGNIFICANT
nb.bin.Tmax <- glm.nb(Events.sum ~ Tmax.avg+ offset(log(visitors.sum)), data = dfB) #SIGNIFICANT
nb.bin.RH <- glm.nb(Events.sum ~ RHmin.avg+ offset(log(visitors.sum)), data = dfB)#SIGNIFICANT
nb.bin.HI <- glm.nb(Events.sum ~ HI.avg+ offset(log(visitors.sum)), data = dfB)#SIGNIFICANT

#forward selection
nb.bin.init <- glm.nb(Events.sum ~ Tmax.avg + RHmin.avg + as.factor(month) +  offset(log(visitors.sum)), data = dfB)
# anova(nb.bin.init, nb.bin.null, test="LR") #p=9.392709e-12
# anova(nb.bin.init, nb.bin.HI, test="LR") #p=5.934092e-09
# anova(nb.bin.init, nb.bin.month, test="LR") #p=1.670795e-07
# anova(nb.bin.init, nb.bin.RH, test="LR") #p=4.279079e-07

#backward elimination
nb.bin.init1 <- glm.nb(Events.sum ~ Tmax.avg + as.factor(month) +  offset(log(visitors.sum)), data = dfB)
#anova(nb.bin.init, nb.bin.init1, test="LR") #p=0.02600323 --> significantly better
# anova(nb.bin.init1, nb.bin.HI, test="LR") #p=7.289149e-09
# anova(nb.bin.init1, nb.bin.month, test="LR") #p=1.103476e-07

nb.bin.init2 <- glm.nb(Events.sum ~ RHmin.avg + as.factor(month) +  offset(log(visitors.sum)), data = dfB)
#anova(nb.bin.init, nb.bin.init2, test="LR") #p=0.005522939--> significantly better

nb.bin.init3 <- glm.nb(Events.sum ~ Tmax.avg + as.factor(month) +  offset(log(visitors.sum)), data = dfB)
#anova(nb.bin.init, nb.bin.init3, test="LR") #p=0.02600323--> significantly better

#check for interactions
nb.bin.int1 <- glm.nb(Events.sum ~ Tmax.avg + RHmin.avg+ as.factor(month) + HI.avg*as.factor(month)+ offset(log(visitors.sum)), data = dfB) 
#anova(nb.bin.int1, nb.bin.init1, test="LR") #p=0.5002751 --> no better

nb.bin.int2 <- glm.nb(Events.sum ~ Tmax.avg + RHmin.avg+ as.factor(month) + HI.avg*RHmin.avg+ offset(log(visitors.sum)), data = dfB) 
#anova(nb.bin.int2, nb.bin.init1, test="LR") #p=0.1139926 --> no better

nb.bin.int3 <- glm.nb(Events.sum ~ Tmax.avg + RHmin.avg+as.factor(month) + as.factor(month)*RHmin.avg+ offset(log(visitors.sum)), data = dfB)
#anova(nb.bin.int3, nb.bin.init1, test="LR") #p=0.007813018 DOES BETTER

#######################################################################################################
#create poisson models
#######################################################################################################

pois.bin.null <- glm(Events.sum ~ 1 + offset(log(visitors.sum)), family = poisson, data = dfB)

#single variables
pois.bin.wk <-glm(Events.sum ~ week + offset(log(visitors.sum)), family = poisson, data = dfB) #not sign
pois.bin.yr <- glm(Events.sum ~ as.factor(year)+ offset(log(visitors.sum)), family = poisson,data = dfB)#not sign
pois.bin.month <- glm(Events.sum ~ as.factor(month)+ offset(log(visitors.sum)), family = poisson,data = dfB) #SIGNIFICANT
pois.bin.Tmax <- glm(Events.sum ~ Tmax.avg+ offset(log(visitors.sum)), family = poisson,data = dfB) #SIGNIFICANT
pois.bin.RH <- glm(Events.sum ~ RHmin.avg+ offset(log(visitors.sum)), family = poisson, data = dfB)#SIGNIFICANT
pois.bin.HI <- glm(Events.sum ~ HI.avg+ offset(log(visitors.sum)), family = poisson, data = dfB)#SIGNIFICANT

#forward selection
pois.bin.init <- glm(Events.sum ~ Tmax.avg + RHmin.avg + as.factor(month) +  offset(log(visitors.sum)), family = poisson, data = dfB)
# anova(pois.bin.init, pois.bin.null, test="LR") #p=< 2.2e-16
# anova(pois.bin.init, pois.bin.HI, test="LR") #p=9.585e-13 
# anova(pois.bin.init, pois.bin.month, test="LR") #p=1.563e-09

#Backward elimination
pois.bin.init1 <- glm(Events.sum ~ Tmax.avg + as.factor(month) +  offset(log(visitors.sum)), family = poisson, data = dfB)
anova(pois.bin.init, pois.bin.init1, test="LR") #p= 0.05642 -- BETTER
```

```
## Analysis of Deviance Table
## 
## Model 1: Events.sum ~ Tmax.avg + RHmin.avg + as.factor(month) + offset(log(visitors.sum))
## Model 2: Events.sum ~ Tmax.avg + as.factor(month) + offset(log(visitors.sum))
##   Resid. Df Resid. Dev Df Deviance Pr(>Chi)   
## 1       152     205.84                        
## 2       153     213.53 -1  -7.6821 0.005577 **
## ---
## Signif. codes:  0 '***' 0.001 '**' 0.01 '*' 0.05 '.' 0.1 ' ' 1
```

```
pois.bin.init2 <- glm(Events.sum ~ RHmin.avg + as.factor(month) +  offset(log(visitors.sum)), family = poisson, data = dfB)
anova(pois.bin.init, pois.bin.init2, test="LR") #p= 0.002736 -- BETTER
```

```
## Analysis of Deviance Table
## 
## Model 1: Events.sum ~ Tmax.avg + RHmin.avg + as.factor(month) + offset(log(visitors.sum))
## Model 2: Events.sum ~ RHmin.avg + as.factor(month) + offset(log(visitors.sum))
##   Resid. Df Resid. Dev Df Deviance Pr(>Chi)   
## 1       152     205.84                        
## 2       153     213.84 -1  -7.9995 0.004679 **
## ---
## Signif. codes:  0 '***' 0.001 '**' 0.01 '*' 0.05 '.' 0.1 ' ' 1
```

```
pois.bin.init3 <- glm(Events.sum ~ Tmax.avg + RHmin.avg +  offset(log(visitors.sum)), family = poisson, data = dfB)
anova(pois.bin.init, pois.bin.init3, test="LR") #p= 5.579e-06  -- BETTER
```

```
## Analysis of Deviance Table
## 
## Model 1: Events.sum ~ Tmax.avg + RHmin.avg + as.factor(month) + offset(log(visitors.sum))
## Model 2: Events.sum ~ Tmax.avg + RHmin.avg + offset(log(visitors.sum))
##   Resid. Df Resid. Dev Df Deviance  Pr(>Chi)    
## 1       152     205.84                          
## 2       157     236.45 -5  -30.611 1.118e-05 ***
## ---
## Signif. codes:  0 '***' 0.001 '**' 0.01 '*' 0.05 '.' 0.1 ' ' 1
```

```
#check for interactions
pois.bin.int1 <- glm(Events.sum ~ Tmax.avg + RHmin.avg + as.factor(month) + HI.avg*as.factor(month)+ offset(log(visitors.sum)), family= poisson, data = dfB) 
# anova(pois.bin.int1, pois.bin.init1, test="LR") #p=0.2156 --> no better

pois.bin.int2 <- glm(Events.sum ~ Tmax.avg + RHmin.avg + as.factor(month) + HI.avg*RHmin.avg+ offset(log(visitors.sum)), family= poisson, data = dfB) 
# anova(pois.bin.int2, pois.bin.init1, test="LR") #p=0.2109 --> no better

pois.bin.int3 <- glm(Events.sum ~ Tmax.avg + RHmin.avg + as.factor(month) + RHmin.avg*as.factor(month)+ offset(log(visitors.sum)), family= poisson, data = dfB) 
# anova(pois.bin.int3, pois.bin.init1, test="LR") #p=0.0165 --> BETTER
# anova(pois.bin.int3, pois.bin.init, test="LR") #p=0.0.0363  --> BETTER


#Format
df.bin2 <- data.frame("Model" = c("nb.bin.null", "nb.bin.wk", "nb.bin.yr", 
                                 "nb.bin.month", "nb.bin.Tmax", "nb.bin.RH", 
                                 "nb.bin.HI", "nb.bin.init",
                                 "nb.bin.init1", "nb.bin.init2", "nb.bin.init3", 
                                 "nb.bin.int1","nb.bin.int2","nb.bin.int3",
                                 "pois.bin.null", "pois.bin.wk", "pois.bin.yr", 
                                 "pois.bin.month", "pois.bin.Tmax", "pois.bin.RH", 
                                 "pois.bin.HI", "pois.bin.init", 
                                 "pois.bin.init1", "pois.bin.init2", "pois.bin.init3", 
                                 "pois.bin.int1", "pois.bin.int2", "pois.bin.int3"),
                      "AIC" = c(nb.bin.null$aic, nb.bin.wk$aic, nb.bin.yr$aic, 
                                nb.bin.month$aic, nb.bin.Tmax$aic, nb.bin.RH$aic, 
                                nb.bin.HI$aic, nb.bin.init$aic, 
                                nb.bin.init1$aic,nb.bin.init2$aic,nb.bin.init3$aic, 
                                nb.bin.int1$aic,nb.bin.int1$aic,nb.bin.int1$aic,
                                pois.bin.null$aic, pois.bin.wk$aic, pois.bin.yr$aic, 
                                pois.bin.month$aic, pois.bin.Tmax$aic, pois.bin.RH$aic, 
                                pois.bin.HI$aic, pois.bin.init$aic, 
                                pois.bin.init1$aic, pois.bin.init2$aic, pois.bin.init3$aic, 
                                pois.bin.int1$aic, pois.bin.int2$aic, pois.bin.int3$aic),
                      "Deviance" = c(nb.bin.null$deviance, nb.bin.wk$deviance, nb.bin.yr$deviance, 
                                     nb.bin.month$deviance, nb.bin.Tmax$deviance, nb.bin.RH$deviance, 
                                     nb.bin.HI$deviance, nb.bin.init$deviance, 
                                     nb.bin.init1$deviance, nb.bin.init2$deviance, nb.bin.init3$deviance, 
                                     nb.bin.int1$deviance, nb.bin.int2$deviance, nb.bin.int3$deviance,
                                     pois.bin.null$deviance, pois.bin.wk$deviance, pois.bin.yr$deviance, 
                                     pois.bin.month$deviance, pois.bin.Tmax$deviance, pois.bin.RH$deviance, 
                                     pois.bin.HI$deviance, pois.bin.init$deviance, 
                                     pois.bin.init1$deviance,pois.bin.init2$deviance,pois.bin.init3$deviance, 
                                     pois.bin.int1$deviance, pois.bin.int2$deviance, pois.bin.int3$deviance),
                      "CV"= c(boot::cv.glm(dfB, nb.bin.null, K = 10)$delta[[2]],
                              boot::cv.glm(dfB, nb.bin.wk, K = 10)$delta[[2]],
                              boot::cv.glm(dfB, nb.bin.yr, K = 10)$delta[[2]],
                              boot::cv.glm(dfB, nb.bin.month, K = 10)$delta[[2]],
                              boot::cv.glm(dfB, nb.bin.Tmax, K = 10)$delta[[2]],
                              boot::cv.glm(dfB, nb.bin.RH, K = 10)$delta[[2]],
                              boot::cv.glm(dfB, nb.bin.HI, K = 10)$delta[[2]],
                              boot::cv.glm(dfB, nb.bin.init, K = 10)$delta[[2]],
                              boot::cv.glm(dfB, nb.bin.init1, K = 10)$delta[[2]],
                              boot::cv.glm(dfB, nb.bin.init2, K = 10)$delta[[2]],
                              boot::cv.glm(dfB, nb.bin.init3, K = 10)$delta[[2]],
                              boot::cv.glm(dfB, nb.bin.int1, K = 10)$delta[[2]],
                              boot::cv.glm(dfB, nb.bin.int2, K = 10)$delta[[2]],
                              boot::cv.glm(dfB, nb.bin.int3, K = 10)$delta[[2]],
                              boot::cv.glm(dfB, pois.bin.null, K = 10)$delta[[2]],
                              boot::cv.glm(dfB, pois.bin.wk, K = 10)$delta[[2]],
                              boot::cv.glm(dfB, pois.bin.yr, K = 10)$delta[[2]],
                              boot::cv.glm(dfB, pois.bin.month, K = 10)$delta[[2]],
                              boot::cv.glm(dfB, pois.bin.Tmax, K = 10)$delta[[2]],
                              boot::cv.glm(dfB, pois.bin.RH, K = 10)$delta[[2]],
                              boot::cv.glm(dfB, pois.bin.HI, K = 10)$delta[[2]],
                              boot::cv.glm(dfB, pois.bin.init, K = 10)$delta[[2]],                              
                              boot::cv.glm(dfB, pois.bin.init1, K = 10)$delta[[2]],
                              boot::cv.glm(dfB, pois.bin.init2, K = 10)$delta[[2]],
                              boot::cv.glm(dfB, pois.bin.init3, K = 10)$delta[[2]],
                              boot::cv.glm(dfB, pois.bin.int1, K = 10)$delta[[2]],
                              boot::cv.glm(dfB, pois.bin.int2, K = 10)$delta[[2]],
                              boot::cv.glm(dfB, pois.bin.int3, K = 10)$delta[[2]]))
```

```
## Warning in theta.ml(Y, mu, sum(w), w, limit = control$maxit, trace =
## control$trace > : iteration limit reached

## Warning in theta.ml(Y, mu, sum(w), w, limit = control$maxit, trace =
## control$trace > : iteration limit reached

## Warning in theta.ml(Y, mu, sum(w), w, limit = control$maxit, trace =
## control$trace > : iteration limit reached

## Warning in theta.ml(Y, mu, sum(w), w, limit = control$maxit, trace =
## control$trace > : iteration limit reached

## Warning in theta.ml(Y, mu, sum(w), w, limit = control$maxit, trace =
## control$trace > : iteration limit reached

## Warning in theta.ml(Y, mu, sum(w), w, limit = control$maxit, trace =
## control$trace > : iteration limit reached

## Warning in theta.ml(Y, mu, sum(w), w, limit = control$maxit, trace =
## control$trace > : iteration limit reached

## Warning in theta.ml(Y, mu, sum(w), w, limit = control$maxit, trace =
## control$trace > : iteration limit reached

## Warning in theta.ml(Y, mu, sum(w), w, limit = control$maxit, trace =
## control$trace > : iteration limit reached

## Warning in theta.ml(Y, mu, sum(w), w, limit = control$maxit, trace =
## control$trace > : iteration limit reached

## Warning in theta.ml(Y, mu, sum(w), w, limit = control$maxit, trace =
## control$trace > : iteration limit reached

## Warning in theta.ml(Y, mu, sum(w), w, limit = control$maxit, trace =
## control$trace > : iteration limit reached

## Warning in theta.ml(Y, mu, sum(w), w, limit = control$maxit, trace =
## control$trace > : iteration limit reached

## Warning in theta.ml(Y, mu, sum(w), w, limit = control$maxit, trace =
## control$trace > : iteration limit reached

## Warning in theta.ml(Y, mu, sum(w), w, limit = control$maxit, trace =
## control$trace > : iteration limit reached

## Warning in theta.ml(Y, mu, sum(w), w, limit = control$maxit, trace =
## control$trace > : iteration limit reached

## Warning in theta.ml(Y, mu, sum(w), w, limit = control$maxit, trace =
## control$trace > : iteration limit reached

## Warning in theta.ml(Y, mu, sum(w), w, limit = control$maxit, trace =
## control$trace > : iteration limit reached

## Warning in theta.ml(Y, mu, sum(w), w, limit = control$maxit, trace =
## control$trace > : iteration limit reached

## Warning in theta.ml(Y, mu, sum(w), w, limit = control$maxit, trace =
## control$trace > : iteration limit reached

## Warning in theta.ml(Y, mu, sum(w), w, limit = control$maxit, trace =
## control$trace > : iteration limit reached

## Warning in theta.ml(Y, mu, sum(w), w, limit = control$maxit, trace =
## control$trace > : iteration limit reached

## Warning in theta.ml(Y, mu, sum(w), w, limit = control$maxit, trace =
## control$trace > : iteration limit reached

## Warning in theta.ml(Y, mu, sum(w), w, limit = control$maxit, trace =
## control$trace > : iteration limit reached

## Warning in theta.ml(Y, mu, sum(w), w, limit = control$maxit, trace =
## control$trace > : iteration limit reached

## Warning in theta.ml(Y, mu, sum(w), w, limit = control$maxit, trace =
## control$trace > : iteration limit reached
```

```
## Warning in glm.nb(formula = Events.sum ~ Tmax.avg + RHmin.avg + as.factor(month)
## + : alternation limit reached
```

## Cross validate

Heat index models:

```
#check
set.seed(2357)
dfB$train <- sample(100, size = nrow(dfB), replace = TRUE)
dfB$train <- ifelse(dfB$train > 70, 0, 1)

#train prediction
nb.bin.null.train <- glm.nb(Events.sum ~ 1 + offset(log(visitors.sum)), data = dfB%>%filter(train==1))
nb.bin.wk.train <-glm.nb(Events.sum ~ week + offset(log(visitors.sum)), data = dfB%>%filter(train==1)) 
nb.bin.yr.train <- glm.nb(Events.sum ~ as.factor(year)+ offset(log(visitors.sum)), 
                          data = dfB%>%filter(train==1))
nb.bin.month.train <- glm.nb(Events.sum ~ as.factor(month)+ offset(log(visitors.sum)), 
                             data = dfB%>%filter(train==1)) 
nb.bin.Tmax.train <- glm.nb(Events.sum ~ Tmax.avg+ offset(log(visitors.sum)), data = dfB%>%filter(train==1)) 
nb.bin.RH.train <- glm.nb(Events.sum ~ RHmin.avg+ offset(log(visitors.sum)), data = dfB%>%filter(train==1))
nb.bin.HI.train <- glm.nb(Events.sum ~ HI.avg+ offset(log(visitors.sum)), data = dfB%>%filter(train==1))
nb.bin.init.train <- glm.nb(Events.sum ~ HI.avg + as.factor(month) +  offset(log(visitors.sum)), 
                            data = dfB%>%filter(train==1))
nb.bin.int.train <- glm.nb(Events.sum ~ HI.avg + as.factor(month) + HI.avg*as.factor(month)+
                       offset(log(visitors.sum)), data = dfB%>%filter(train==1)) 
pois.bin.null.train <- glm(Events.sum ~ 1 + offset(log(visitors.sum)), family = poisson, 
                           data = dfB%>%filter(train==1))
pois.bin.wk.train <-glm(Events.sum ~ week + offset(log(visitors.sum)), family = poisson, 
                        data = dfB%>%filter(train==1)) 
pois.bin.yr.train <- glm(Events.sum ~ as.factor(year)+ offset(log(visitors.sum)), 
                         family = poisson,data = dfB%>%filter(train==1)) 
pois.bin.month.train <- glm(Events.sum ~ as.factor(month)+ offset(log(visitors.sum)), 
                            family = poisson,data = dfB%>%filter(train==1)) 
pois.bin.Tmax.train <- glm(Events.sum ~ Tmax.avg+ offset(log(visitors.sum)), family = poisson,
                           data = dfB%>%filter(train==1)) 
pois.bin.RH.train <- glm(Events.sum ~ RHmin.avg+ offset(log(visitors.sum)), family = poisson, 
                         data = dfB%>%filter(train==1))
pois.bin.HI.train <- glm(Events.sum ~ HI.avg+ offset(log(visitors.sum)), family = poisson, 
                         data = dfB%>%filter(train==1))
pois.bin.init.train <- glm(Events.sum ~ HI.avg + as.factor(month) +  offset(log(visitors.sum)), 
                     family = poisson, data = dfB%>%filter(train==1))
pois.bin.int.train <- glm(Events.sum ~ HI.avg + as.factor(month) + HI.avg*as.factor(month)+
                      offset(log(visitors.sum)), family= poisson, data = dfB%>%filter(train==1)) 


#apply prediction
pred.train <- dfB%>%filter(train==0) %>%
  mutate(nb.null      = predict(nb.bin.null.train , newdata=dfB%>%filter(train==0),type='response'),
         nb.week      = predict(nb.bin.wk.train, newdata=dfB%>%filter(train==0),type='response'),
         nb.year      = predict(nb.bin.yr.train, newdata=dfB%>%filter(train==0),type='response'),
         nb.month     = predict(nb.bin.month.train, newdata=dfB%>%filter(train==0),type='response'),
         nb.Tmax      = predict(nb.bin.Tmax.train, newdata=dfB%>%filter(train==0),type='response'),
         nb.RH        = predict(nb.bin.RH.train, newdata=dfB%>%filter(train==0),type='response'),
         nb.HI        = predict(nb.bin.HI.train, newdata=dfB%>%filter(train==0),type='response'),
         nb.init      = predict(nb.bin.init.train, newdata=dfB%>%filter(train==0),type='response'),
         nb.int       = predict(nb.bin.init.train, newdata=dfB%>%filter(train==0),type='response'),
         pois.null    = predict(pois.bin.null.train , newdata=dfB%>%filter(train==0),type='response'),
         pois.week    = predict(pois.bin.wk.train, newdata=dfB%>%filter(train==0),type='response'),
         pois.year    = predict(pois.bin.yr.train, newdata=dfB%>%filter(train==0),type='response'),
         pois.month   = predict(pois.bin.month.train, newdata=dfB%>%filter(train==0),type='response'),
         pois.Tmax    = predict(pois.bin.Tmax.train, newdata=dfB%>%filter(train==0),type='response'),
         pois.RH      = predict(pois.bin.RH.train, newdata=dfB%>%filter(train==0),type='response'),
         pois.HI      = predict(pois.bin.HI.train, newdata=dfB%>%filter(train==0),type='response'),
         pois.init    = predict(pois.bin.init.train, newdata=dfB%>%filter(train==0),type='response'),
         pois.int     = predict(pois.bin.int.train, newdata=dfB%>%filter(train==0),type='response'))


df.bin$train.MSE <- c(MLmetrics::MSE(pred.train$Events.sum, pred.train$nb.null),
                       MLmetrics::MSE(pred.train$Events.sum, pred.train$nb.week),
                       MLmetrics::MSE(pred.train$Events.sum, pred.train$nb.year),
                       MLmetrics::MSE(pred.train$Events.sum, pred.train$nb.month),
                       MLmetrics::MSE(pred.train$Events.sum, pred.train$nb.Tmax),
                       MLmetrics::MSE(pred.train$Events.sum, pred.train$nb.RH),
                       MLmetrics::MSE(pred.train$Events.sum, pred.train$nb.HI),
                       MLmetrics::MSE(pred.train$Events.sum, pred.train$nb.init),
                       MLmetrics::MSE(pred.train$Events.sum, pred.train$nb.int),
                       MLmetrics::MSE(pred.train$Events.sum, pred.train$pois.null),
                       MLmetrics::MSE(pred.train$Events.sum, pred.train$pois.week),
                       MLmetrics::MSE(pred.train$Events.sum, pred.train$pois.year),
                       MLmetrics::MSE(pred.train$Events.sum, pred.train$pois.month),
                       MLmetrics::MSE(pred.train$Events.sum, pred.train$pois.Tmax),
                       MLmetrics::MSE(pred.train$Events.sum, pred.train$pois.RH),
                       MLmetrics::MSE(pred.train$Events.sum, pred.train$pois.HI),
                       MLmetrics::MSE(pred.train$Events.sum, pred.train$pois.init),
                       MLmetrics::MSE(pred.train$Events.sum, pred.train$pois.int))
             

#apply regressions to test data
pred.test <- dfB%>%filter(train==1) %>%
  mutate(nb.null      = predict(nb.bin.null.train , newdata=dfB%>%filter(train==1),type='response'),
         nb.week      = predict(nb.bin.wk.train, newdata=dfB%>%filter(train==1),type='response'),
         nb.year      = predict(nb.bin.yr.train, newdata=dfB%>%filter(train==1),type='response'),
         nb.month     = predict(nb.bin.month.train, newdata=dfB%>%filter(train==1),type='response'),
         nb.Tmax      = predict(nb.bin.Tmax.train, newdata=dfB%>%filter(train==1),type='response'),
         nb.RH        = predict(nb.bin.RH.train, newdata=dfB%>%filter(train==1),type='response'),
         nb.HI        = predict(nb.bin.HI.train, newdata=dfB%>%filter(train==1),type='response'),
         nb.init      = predict(nb.bin.init.train, newdata=dfB%>%filter(train==1),type='response'),
         nb.int       = predict(nb.bin.init.train, newdata=dfB%>%filter(train==1),type='response'),
         pois.null    = predict(pois.bin.null.train , newdata=dfB%>%filter(train==1),type='response'),
         pois.week    = predict(pois.bin.wk.train, newdata=dfB%>%filter(train==1),type='response'),
         pois.year    = predict(pois.bin.yr.train, newdata=dfB%>%filter(train==1),type='response'),
         pois.month   = predict(pois.bin.month.train, newdata=dfB%>%filter(train==1),type='response'),
         pois.Tmax    = predict(pois.bin.Tmax.train, newdata=dfB%>%filter(train==1),type='response'),
         pois.RH      = predict(pois.bin.RH.train, newdata=dfB%>%filter(train==1),type='response'),
         pois.HI      = predict(pois.bin.HI.train, newdata=dfB%>%filter(train==1),type='response'),
         pois.init    = predict(pois.bin.init.train, newdata=dfB%>%filter(train==1),type='response'),
         pois.int     = predict(pois.bin.int.train, newdata=dfB%>%filter(train==1),type='response'))


df.bin$test.MSE <- c(MLmetrics::MSE(pred.test$Events.sum, pred.test$nb.null),
                       MLmetrics::MSE(pred.test$Events.sum, pred.test$nb.week),
                       MLmetrics::MSE(pred.test$Events.sum, pred.test$nb.year),
                       MLmetrics::MSE(pred.test$Events.sum, pred.test$nb.month),
                       MLmetrics::MSE(pred.test$Events.sum, pred.test$nb.Tmax),
                       MLmetrics::MSE(pred.test$Events.sum, pred.test$nb.RH),
                       MLmetrics::MSE(pred.test$Events.sum, pred.test$nb.HI),
                       MLmetrics::MSE(pred.test$Events.sum, pred.test$nb.init),
                       MLmetrics::MSE(pred.test$Events.sum, pred.test$nb.int),
                       MLmetrics::MSE(pred.test$Events.sum, pred.test$pois.null),
                       MLmetrics::MSE(pred.test$Events.sum, pred.test$pois.week),
                       MLmetrics::MSE(pred.test$Events.sum, pred.test$pois.year),
                       MLmetrics::MSE(pred.test$Events.sum, pred.test$pois.month),
                       MLmetrics::MSE(pred.test$Events.sum, pred.test$pois.Tmax),
                       MLmetrics::MSE(pred.test$Events.sum, pred.test$pois.RH),
                       MLmetrics::MSE(pred.test$Events.sum, pred.test$pois.HI),
                       MLmetrics::MSE(pred.test$Events.sum, pred.test$pois.init),
                       MLmetrics::MSE(pred.test$Events.sum, pred.test$pois.int))

df.bin
```

```
##             Model      AIC Deviance       CV train.MSE test.MSE
## 1     nb.bin.null 678.4256 179.5117 5.659663  6.098491 5.364810
## 2       nb.bin.wk 680.0663 179.5062 5.631151  6.070742 5.359909
## 3       nb.bin.yr 686.4338 178.8409 6.168251  6.386862 5.310848
## 4    nb.bin.month 648.0225 177.2028 4.788908  6.025884 4.144541
## 5     nb.bin.Tmax 658.1899 176.9261 5.119245  5.008651 5.044169
## 6       nb.bin.RH 652.5287 181.9941 4.606962  4.600756 4.615422
## 7       nb.bin.HI 663.4226 178.8688 5.161095  5.208839 5.062292
## 8     nb.bin.init 634.5005 180.3237 4.187208  5.890508 3.437152
## 9      nb.bin.int 638.2262 178.8587 4.339646  5.890508 3.437152
## 10  pois.bin.null 710.9170 304.4028 5.659663  6.106445 5.364199
## 11    pois.bin.wk 712.2627 303.7485 5.631151  6.078569 5.359339
## 12    pois.bin.yr 718.1520 301.6378 6.168251  6.503346 5.291208
## 13 pois.bin.month 657.5659 241.0517 4.788908  6.026940 4.144328
## 14  pois.bin.Tmax 677.0876 268.5734 5.119245  5.044360 5.036774
## 15    pois.bin.RH 664.5303 256.0161 4.606962  4.585100 4.613134
## 16    pois.bin.HI 684.0665 275.5522 5.161095  5.227874 5.054729
## 17  pois.bin.init 637.0046 218.4904 4.187208  5.915004 3.434222
## 18   pois.bin.int 639.5408 211.0265 4.339646  6.645427 3.311304
```

**Take away: nb init model does much better than the null model**

Tmax/RHmin models

```
#check
set.seed(2357)
dfB$train <- sample(100, size = nrow(dfB), replace = TRUE)
dfB$train <- ifelse(dfB$train > 70, 0, 1)

#train prediction
nb.bin.null.train <- glm.nb(Events.sum ~ 1 + offset(log(visitors.sum)), data = dfB%>%filter(train==1))
nb.bin.wk.train <-glm.nb(Events.sum ~ week + offset(log(visitors.sum)), data = dfB%>%filter(train==1)) 
nb.bin.yr.train <- glm.nb(Events.sum ~ as.factor(year)+ offset(log(visitors.sum)), 
                          data = dfB%>%filter(train==1))
nb.bin.month.train <- glm.nb(Events.sum ~ as.factor(month)+ offset(log(visitors.sum)), 
                             data = dfB%>%filter(train==1)) 
nb.bin.Tmax.train <- glm.nb(Events.sum ~ Tmax.avg+ offset(log(visitors.sum)), data = dfB%>%filter(train==1)) 
nb.bin.RH.train <- glm.nb(Events.sum ~ RHmin.avg+ offset(log(visitors.sum)), data = dfB%>%filter(train==1))
nb.bin.HI.train <- glm.nb(Events.sum ~ HI.avg+ offset(log(visitors.sum)), data = dfB%>%filter(train==1))
nb.bin.init.train <- glm.nb(Events.sum ~ Tmax.avg + RHmin.avg+ as.factor(month) +  offset(log(visitors.sum)), 
                            data = dfB%>%filter(train==1))
nb.bin.init1.train <- glm.nb(Events.sum ~ Tmax.avg + as.factor(month) +  offset(log(visitors.sum)), 
                             data = dfB%>%filter(train==1))
nb.bin.init2.train <- glm.nb(Events.sum ~ RHmin.avg + as.factor(month) +  offset(log(visitors.sum)), 
                             data = dfB%>%filter(train==1))
nb.bin.init3.train <- glm.nb(Events.sum ~ Tmax.avg + RHmin.avg +  offset(log(visitors.sum)), 
                             data = dfB%>%filter(train==1))
nb.bin.int1.train <- glm.nb(Events.sum ~ Tmax.avg+ RHmin.avg + as.factor(month) + HI.avg*as.factor(month)+
                              offset(log(visitors.sum)), data = dfB%>%filter(train==1)) 
nb.bin.int2.train <- glm.nb(Events.sum ~ Tmax.avg + RHmin.avg+ as.factor(month) + HI.avg*RHmin.avg+
                              offset(log(visitors.sum)), data = dfB%>%filter(train==1)) 
nb.bin.int3.train <- glm.nb(Events.sum ~ Tmax.avg + RHmin.avg+ as.factor(month) + RHmin.avg*as.factor(month)+
                              offset(log(visitors.sum)), data = dfB%>%filter(train==1)) 
pois.bin.null.train <- glm(Events.sum ~ 1 + offset(log(visitors.sum)), family = poisson, 
                           data = dfB%>%filter(train==1))
pois.bin.wk.train <-glm(Events.sum ~ week + offset(log(visitors.sum)), family = poisson, 
                        data = dfB%>%filter(train==1)) 
pois.bin.yr.train <- glm(Events.sum ~ as.factor(year)+ offset(log(visitors.sum)), 
                         family = poisson,data = dfB%>%filter(train==1)) 
pois.bin.month.train <- glm(Events.sum ~ as.factor(month)+ offset(log(visitors.sum)), 
                            family = poisson,data = dfB%>%filter(train==1)) 
pois.bin.Tmax.train <- glm(Events.sum ~ Tmax.avg+ offset(log(visitors.sum)), family = poisson,
                           data = dfB%>%filter(train==1)) 
pois.bin.RH.train <- glm(Events.sum ~ RHmin.avg+ offset(log(visitors.sum)), family = poisson, 
                         data = dfB%>%filter(train==1))
pois.bin.HI.train <- glm(Events.sum ~ HI.avg+ offset(log(visitors.sum)), family = poisson, 
                         data = dfB%>%filter(train==1))
pois.bin.init.train <- glm(Events.sum ~ Tmax.avg + RHmin.avg+ as.factor(month) +  offset(log(visitors.sum)), 
                           family = poisson, data = dfB%>%filter(train==1))
pois.bin.init1.train <- glm(Events.sum ~ Tmax.avg + as.factor(month) +  offset(log(visitors.sum)), 
                            family = poisson, data = dfB%>%filter(train==1))
pois.bin.init2.train <- glm(Events.sum ~ RHmin.avg + as.factor(month) +  offset(log(visitors.sum)), 
                            family = poisson, data = dfB%>%filter(train==1))
pois.bin.init3.train <- glm(Events.sum ~ Tmax.avg + RHmin.avg +  offset(log(visitors.sum)), 
                            family = poisson, data = dfB%>%filter(train==1))
pois.bin.int1.train <- glm(Events.sum ~ Tmax.avg + RHmin.avg+ as.factor(month) + HI.avg*as.factor(month)+
                             offset(log(visitors.sum)), family= poisson, data = dfB%>%filter(train==1))
pois.bin.int2.train <- glm(Events.sum ~ Tmax.avg + RHmin.avg+ as.factor(month) + HI.avg*RHmin.avg+
                             offset(log(visitors.sum)), family= poisson, data = dfB%>%filter(train==1))
pois.bin.int3.train <- glm(Events.sum ~ Tmax.avg + RHmin.avg+ as.factor(month) + RHmin.avg*as.factor(month)+
                             offset(log(visitors.sum)), family= poisson, data = dfB%>%filter(train==1))
#apply prediction
pred.train <- dfB%>%filter(train==0) %>%
  mutate(nb.null      = predict(nb.bin.null.train , newdata=dfB%>%filter(train==0),type='response'),
         nb.week      = predict(nb.bin.wk.train, newdata=dfB%>%filter(train==0),type='response'),
         nb.year      = predict(nb.bin.yr.train, newdata=dfB%>%filter(train==0),type='response'),
         nb.month     = predict(nb.bin.month.train, newdata=dfB%>%filter(train==0),type='response'),
         nb.Tmax      = predict(nb.bin.Tmax.train, newdata=dfB%>%filter(train==0),type='response'),
         nb.RH        = predict(nb.bin.RH.train, newdata=dfB%>%filter(train==0),type='response'),
         nb.HI        = predict(nb.bin.HI.train, newdata=dfB%>%filter(train==0),type='response'),
         nb.init      = predict(nb.bin.init.train, newdata=dfB%>%filter(train==0),type='response'),
         nb.init1     = predict(nb.bin.init1.train, newdata=dfB%>%filter(train==0),type='response'),
         nb.init2     = predict(nb.bin.init2.train, newdata=dfB%>%filter(train==0),type='response'),
         nb.init3     = predict(nb.bin.init3.train, newdata=dfB%>%filter(train==0),type='response'),
         nb.int1      = predict(nb.bin.int1.train, newdata=dfB%>%filter(train==0),type='response'),
         nb.int2      = predict(nb.bin.int2.train, newdata=dfB%>%filter(train==0),type='response'),
         nb.int3      = predict(nb.bin.int3.train, newdata=dfB%>%filter(train==0),type='response'),
         pois.null    = predict(pois.bin.null.train , newdata=dfB%>%filter(train==0),type='response'),
         pois.week    = predict(pois.bin.wk.train, newdata=dfB%>%filter(train==0),type='response'),
         pois.year    = predict(pois.bin.yr.train, newdata=dfB%>%filter(train==0),type='response'),
         pois.month   = predict(pois.bin.month.train, newdata=dfB%>%filter(train==0),type='response'),
         pois.Tmax    = predict(pois.bin.Tmax.train, newdata=dfB%>%filter(train==0),type='response'),
         pois.RH      = predict(pois.bin.RH.train, newdata=dfB%>%filter(train==0),type='response'),
         pois.HI      = predict(pois.bin.HI.train, newdata=dfB%>%filter(train==0),type='response'),
         pois.init    = predict(pois.bin.init.train, newdata=dfB%>%filter(train==0),type='response'),
         pois.init1   = predict(pois.bin.init1.train, newdata=dfB%>%filter(train==0),type='response'),
         pois.init2   = predict(pois.bin.init2.train, newdata=dfB%>%filter(train==0),type='response'),
         pois.init3   = predict(pois.bin.init3.train, newdata=dfB%>%filter(train==0),type='response'),
         pois.int1    = predict(pois.bin.int1.train, newdata=dfB%>%filter(train==0),type='response'),
         pois.int2    = predict(pois.bin.int2.train, newdata=dfB%>%filter(train==0),type='response'),
         pois.int3    = predict(pois.bin.int3.train, newdata=dfB%>%filter(train==0),type='response'))


df.bin2$train.MSE <- c(MLmetrics::MSE(pred.train$Events.sum, pred.train$nb.null),
                       MLmetrics::MSE(pred.train$Events.sum, pred.train$nb.week),
                       MLmetrics::MSE(pred.train$Events.sum, pred.train$nb.year),
                       MLmetrics::MSE(pred.train$Events.sum, pred.train$nb.month),
                       MLmetrics::MSE(pred.train$Events.sum, pred.train$nb.Tmax),
                       MLmetrics::MSE(pred.train$Events.sum, pred.train$nb.RH),
                       MLmetrics::MSE(pred.train$Events.sum, pred.train$nb.HI),
                       MLmetrics::MSE(pred.train$Events.sum, pred.train$nb.init),
                       MLmetrics::MSE(pred.train$Events.sum, pred.train$nb.init1),
                       MLmetrics::MSE(pred.train$Events.sum, pred.train$nb.init2),
                       MLmetrics::MSE(pred.train$Events.sum, pred.train$nb.init3),
                       MLmetrics::MSE(pred.train$Events.sum, pred.train$nb.int1),
                       MLmetrics::MSE(pred.train$Events.sum, pred.train$nb.int2),
                       MLmetrics::MSE(pred.train$Events.sum, pred.train$nb.int3),
                       MLmetrics::MSE(pred.train$Events.sum, pred.train$pois.null),
                       MLmetrics::MSE(pred.train$Events.sum, pred.train$pois.week),
                       MLmetrics::MSE(pred.train$Events.sum, pred.train$pois.year),
                       MLmetrics::MSE(pred.train$Events.sum, pred.train$pois.month),
                       MLmetrics::MSE(pred.train$Events.sum, pred.train$pois.Tmax),
                       MLmetrics::MSE(pred.train$Events.sum, pred.train$pois.RH),
                       MLmetrics::MSE(pred.train$Events.sum, pred.train$pois.HI),
                       MLmetrics::MSE(pred.train$Events.sum, pred.train$pois.init),
                       MLmetrics::MSE(pred.train$Events.sum, pred.train$pois.init1),
                       MLmetrics::MSE(pred.train$Events.sum, pred.train$pois.init2),
                       MLmetrics::MSE(pred.train$Events.sum, pred.train$pois.init3),
                       MLmetrics::MSE(pred.train$Events.sum, pred.train$pois.int1), 
                       MLmetrics::MSE(pred.train$Events.sum, pred.train$pois.int2),
                       MLmetrics::MSE(pred.train$Events.sum, pred.train$pois.int3))


#apply regressions to test data
pred.test <- dfB%>%filter(train==1) %>%
  mutate(nb.null      = predict(nb.bin.null.train , newdata=dfB%>%filter(train==1),type='response'),
         nb.week      = predict(nb.bin.wk.train, newdata=dfB%>%filter(train==1),type='response'),
         nb.year      = predict(nb.bin.yr.train, newdata=dfB%>%filter(train==1),type='response'),
         nb.month     = predict(nb.bin.month.train, newdata=dfB%>%filter(train==1),type='response'),
         nb.Tmax      = predict(nb.bin.Tmax.train, newdata=dfB%>%filter(train==1),type='response'),
         nb.RH        = predict(nb.bin.RH.train, newdata=dfB%>%filter(train==1),type='response'),
         nb.HI        = predict(nb.bin.HI.train, newdata=dfB%>%filter(train==1),type='response'),
         nb.init      = predict(nb.bin.init.train, newdata=dfB%>%filter(train==1),type='response'),
         nb.init1     = predict(nb.bin.init1.train, newdata=dfB%>%filter(train==1),type='response'),
         nb.init2     = predict(nb.bin.init2.train, newdata=dfB%>%filter(train==1),type='response'),
         nb.init3     = predict(nb.bin.init3.train, newdata=dfB%>%filter(train==1),type='response'),
         nb.int1      = predict(nb.bin.int1.train, newdata=dfB%>%filter(train==1),type='response'),
         nb.int2      = predict(nb.bin.int2.train, newdata=dfB%>%filter(train==1),type='response'),
         nb.int3      = predict(nb.bin.int3.train, newdata=dfB%>%filter(train==1),type='response'),
         pois.null    = predict(pois.bin.null.train , newdata=dfB%>%filter(train==1),type='response'),
         pois.week    = predict(pois.bin.wk.train, newdata=dfB%>%filter(train==1),type='response'),
         pois.year    = predict(pois.bin.yr.train, newdata=dfB%>%filter(train==1),type='response'),
         pois.month   = predict(pois.bin.month.train, newdata=dfB%>%filter(train==1),type='response'),
         pois.Tmax    = predict(pois.bin.Tmax.train, newdata=dfB%>%filter(train==1),type='response'),
         pois.RH      = predict(pois.bin.RH.train, newdata=dfB%>%filter(train==1),type='response'),
         pois.HI      = predict(pois.bin.HI.train, newdata=dfB%>%filter(train==1),type='response'),
         pois.init    = predict(pois.bin.init.train, newdata=dfB%>%filter(train==1),type='response'),
         pois.init1   = predict(pois.bin.init1.train, newdata=dfB%>%filter(train==1),type='response'),
         pois.init2   = predict(pois.bin.init2.train, newdata=dfB%>%filter(train==1),type='response'),
         pois.init3   = predict(pois.bin.init3.train, newdata=dfB%>%filter(train==1),type='response'),
         pois.int1    = predict(pois.bin.int1.train, newdata=dfB%>%filter(train==1),type='response'),
         pois.int2    = predict(pois.bin.int2.train, newdata=dfB%>%filter(train==1),type='response'),
         pois.int3    = predict(pois.bin.int3.train, newdata=dfB%>%filter(train==1),type='response'))


df.bin2$test.MSE <- c(MLmetrics::MSE(pred.test$Events.sum, pred.test$nb.null),
                      MLmetrics::MSE(pred.test$Events.sum, pred.test$nb.week),
                      MLmetrics::MSE(pred.test$Events.sum, pred.test$nb.year),
                      MLmetrics::MSE(pred.test$Events.sum, pred.test$nb.month),
                      MLmetrics::MSE(pred.test$Events.sum, pred.test$nb.Tmax),
                      MLmetrics::MSE(pred.test$Events.sum, pred.test$nb.RH),
                      MLmetrics::MSE(pred.test$Events.sum, pred.test$nb.HI),
                      MLmetrics::MSE(pred.test$Events.sum, pred.test$nb.init),
                      MLmetrics::MSE(pred.test$Events.sum, pred.test$nb.init1),
                      MLmetrics::MSE(pred.test$Events.sum, pred.test$nb.init2),
                      MLmetrics::MSE(pred.test$Events.sum, pred.test$nb.init3),
                      MLmetrics::MSE(pred.test$Events.sum, pred.test$nb.int1),
                      MLmetrics::MSE(pred.test$Events.sum, pred.test$nb.int2),
                      MLmetrics::MSE(pred.test$Events.sum, pred.test$nb.int3),
                      MLmetrics::MSE(pred.test$Events.sum, pred.test$pois.null),
                      MLmetrics::MSE(pred.test$Events.sum, pred.test$pois.week),
                      MLmetrics::MSE(pred.test$Events.sum, pred.test$pois.year),
                      MLmetrics::MSE(pred.test$Events.sum, pred.test$pois.month),
                      MLmetrics::MSE(pred.test$Events.sum, pred.test$pois.Tmax),
                      MLmetrics::MSE(pred.test$Events.sum, pred.test$pois.RH),
                      MLmetrics::MSE(pred.test$Events.sum, pred.test$pois.HI),
                      MLmetrics::MSE(pred.test$Events.sum, pred.test$pois.init),
                      MLmetrics::MSE(pred.test$Events.sum, pred.test$pois.init1),
                      MLmetrics::MSE(pred.test$Events.sum, pred.test$pois.init2),
                      MLmetrics::MSE(pred.test$Events.sum, pred.test$pois.init3),
                      MLmetrics::MSE(pred.test$Events.sum, pred.test$pois.int1),
                      MLmetrics::MSE(pred.test$Events.sum, pred.test$pois.int2),
                      MLmetrics::MSE(pred.test$Events.sum, pred.test$pois.int3))

df.bin2
```

```
##             Model      AIC Deviance       CV train.MSE test.MSE
## 1     nb.bin.null 678.4256 179.5117 5.736056  6.098491 5.364810
## 2       nb.bin.wk 680.0663 179.5062 5.898721  6.070742 5.359909
## 3       nb.bin.yr 686.4338 178.8409 6.124883  6.386862 5.310848
## 4    nb.bin.month 648.0225 177.2028 4.818666  6.025884 4.144541
## 5     nb.bin.Tmax 658.1899 176.9261 5.153397  5.008651 5.044169
## 6       nb.bin.RH 652.5287 181.9941 4.646732  4.600756 4.615422
## 7       nb.bin.HI 663.4226 178.8688 5.246254  5.208839 5.062292
## 8     nb.bin.init 625.7181 177.4784 4.080691  5.378976 3.403287
## 9    nb.bin.init1 629.6917 176.5649 4.205841  5.727222 3.521620
## 10   nb.bin.init2 630.6512 179.4557 4.066723  5.477646 3.517336
## 11   nb.bin.init3 629.6917 176.5649 4.161147  3.925469 4.471530
## 12    nb.bin.int1 632.9182 175.3890 4.350486  5.936930 3.241929
## 13    nb.bin.int2 632.9182 176.5345 4.185210  5.415544 3.298029
## 14    nb.bin.int3 632.9182 172.5418 4.230157  5.469912 3.211187
## 15  pois.bin.null 710.9170 304.4028 5.717231  6.106445 5.364199
## 16    pois.bin.wk 712.2627 303.7485 5.674475  6.078569 5.359339
## 17    pois.bin.yr 718.1520 301.6378 6.044483  6.503346 5.291208
## 18 pois.bin.month 657.5659 241.0517 4.818889  6.026940 4.144328
## 19  pois.bin.Tmax 677.0876 268.5734 5.151561  5.044360 5.036774
## 20    pois.bin.RH 664.5303 256.0161 4.711519  4.585100 4.613134
## 21    pois.bin.HI 684.0665 275.5522 5.177428  5.227874 5.054729
## 22  pois.bin.init 626.3575 205.8432 3.978567  5.375114 3.400101
## 23 pois.bin.init1 632.0396 213.5253 4.177075  5.733723 3.519073
## 24 pois.bin.init2 632.3569 213.8427 4.122344  5.474362 3.511615
## 25 pois.bin.init3 646.9685 236.4543 4.515172  3.956620 4.466865
## 26  pois.bin.int1 633.0347 200.5205 4.413647  5.879059 3.229909
## 27  pois.bin.int2 629.6306 205.1164 4.267498  5.424930 3.289379
## 28  pois.bin.int3 623.7249 193.2106 4.098075  5.463421 3.207961
```

**Take away: poisson int3 (HI + RHmin) does better than all the rest model does much better than the null model**

### Visualize best model

```
# dfC$events.pred <-predict(nb.omit_diff, newdata=dfC, type='response')
# dfC$SE <- predict(nb.omit_diff, newdata = dfC, type = 'response',
#                                               se.fit = TRUE)$se.fit
# dfC$upr = dfC$events.pred + (2 * dfC$SE)
# dfC$lwr = dfC$events.pred - (2 * dfC$SE)
# 
# ggplot()+
#   theme_classic()+
#   geom_point(data=dfC, aes(x=Date, y=Events))+
#   geom_line(data=dfC, aes(x=Date, y=events.pred, group = 1), color="red")+
#   #geom_ribbon(data = dfC, aes(x=1:length(Date), ymin = lwr, ymax = upr), alpha = 0.2, fill="red")+
#   ggtitle("Individual days")
# 
# summary(nb.omit_diff)


dfB$events.pred <-predict(pois.bin.int3, newdata=dfB, type='response')
dfB$SE <- predict(pois.bin.int3, newdata = dfB, type = 'response',
                                              se.fit = TRUE)$se.fit
dfB$upr = dfB$events.pred + (2 * dfB$SE)
dfB$lwr = dfB$events.pred - (2 * dfB$SE)

ggplot()+
  theme_classic()+
  geom_point(data=dfB, aes(x=X, y=Events.sum))+
  geom_line(data=dfB, aes(x=X, y=events.pred, group = 1), color="red")+
  #geom_ribbon(data = dfB, aes(x=1:length(X), ymin = lwr, ymax = upr), alpha = 0.2, fill="red")+
  ggtitle("Binned by week")
```

```
#best fit model
summary(pois.bin.int3)
```

```
## 
## Call:
## glm(formula = Events.sum ~ Tmax.avg + RHmin.avg + as.factor(month) + 
##     RHmin.avg * as.factor(month) + offset(log(visitors.sum)), 
##     family = poisson, data = dfB)
## 
## Deviance Residuals: 
##     Min       1Q   Median       3Q      Max  
## -2.5602  -0.8654  -0.1508   0.6168   2.7526  
## 
## Coefficients:
##                              Estimate Std. Error z value Pr(>|z|)    
## (Intercept)                 -11.85301    1.21183  -9.781  < 2e-16 ***
## Tmax.avg                      0.02701    0.01335   2.023  0.04312 *  
## RHmin.avg                    -0.09882    0.04865  -2.031  0.04222 *  
## as.factor(month)5             0.08603    0.74912   0.115  0.90857    
## as.factor(month)6            -0.28481    0.79512  -0.358  0.72019    
## as.factor(month)7            -0.26811    0.77162  -0.347  0.72825    
## as.factor(month)8            -0.88219    0.81047  -1.088  0.27638    
## as.factor(month)9            -2.29704    0.92253  -2.490  0.01278 *  
## RHmin.avg:as.factor(month)5   0.05872    0.05404   1.087  0.27717    
## RHmin.avg:as.factor(month)6   0.03586    0.05842   0.614  0.53932    
## RHmin.avg:as.factor(month)7   0.04375    0.05165   0.847  0.39695    
## RHmin.avg:as.factor(month)8   0.07541    0.05267   1.432  0.15218    
## RHmin.avg:as.factor(month)9   0.17171    0.06004   2.860  0.00424 ** 
## ---
## Signif. codes:  0 '***' 0.001 '**' 0.01 '*' 0.05 '.' 0.1 ' ' 1
## 
## (Dispersion parameter for poisson family taken to be 1)
## 
##     Null deviance: 304.40  on 159  degrees of freedom
## Residual deviance: 193.21  on 147  degrees of freedom
## AIC: 623.72
## 
## Number of Fisher Scoring iterations: 5
```
