# Supplementary material for: Predicting climate-change induced heat-related illness risk in Grand Canyon National Park visitors
Source: PLoS One. 2023 Aug 9;18(8):e0288812. doi: 10.1371/journal.pone.0288812 (PMC10411749; doi:10.1371/journal.pone.0288812)
Supplement: S5 File — Rmd file with code and output for regression model prediction. (HTML) [file pone.0288812.s006.html]

Heat Code Projections


# Heat Code Projections

#### Brinkley Raynor

#### 6/22/2021

# Load and format data

```
#load data
setwd("~/Projects_complete/GRCA_HeatProject")
# dfC <- read.csv("HeatData_Count.csv") 
# dfT <- read.csv("ProjectionData.csv")
#df.grid <- read.csv("GridMet_Clean.csv") 
dfB <- read.csv("HeatData_Week.csv")#binned by week
dfT <- read.csv("ProjectionDataBinned.csv")
df.grid <- read.csv("GridBin_Clean.csv") 

#Look at events per 100k visitors
dfT$visitors.sum <- 100000 
df.grid$visitors.sum <- 100000 

#Subset years of projections
dfP <- subset(dfT, year >= 2018) #only future years
#dfH <- subset(dfT, year < 2018) #only past years
```

Apply model

```
#specify best fit model
model <- glm(Events.sum ~ Tmax.avg + RHmin.avg + as.factor(month) + RHmin.avg*as.factor(month)+ offset(log(visitors.sum)), family= poisson, data = dfB)

#Predict
dfP$pred <- predict(model, dfP, type="response", interval="confidence")
df.grid$pred <-  predict(model, df.grid, type="response", interval="confidence")
dfB$pred <-  predict(model, dfB, type="response", interval="confidence")
dfB$pred <- dfB$pred/dfB$visitors.sum*100000 #weight per 100k visitors
```

Find annual averages

```
#MAcA projections
dfP.wk <- dfP %>%
  dplyr::group_by(GCM, year) %>%
  dplyr::summarise(pred.wk = mean(pred))
dfP.wk$RCP <- str_sub(dfP.wk$GCM, -2, -1)

dfP.wk.45 <- dfP.wk %>% 
  filter(RCP=="45")%>%
  tidyr::pivot_wider(names_from=GCM, values_from=pred.wk)%>% #wide format
  dplyr::select(-RCP)

dfP.wk.85 <- dfP.wk %>% 
  filter(RCP=="85")%>%
  tidyr::pivot_wider(names_from=GCM, values_from=pred.wk)%>% #wide format
  dplyr::select(-RCP)

#Historical grid met data
df.grid.wk <- df.grid %>%
  dplyr::group_by(year) %>%
  dplyr::summarise(pred.wk = mean(pred))

#Observed data
dfB.wk <- dfB %>%
  dplyr::group_by(year) %>%
  dplyr::summarise(pred.wk = mean(pred),
                   real.wk = mean(EventsWeighted),
                   real.LB = t.test(EventsWeighted)$conf.int[[1]],
                   real.UB = t.test(EventsWeighted)$conf.int[[2]])
```

Calculate quantiles

```
#RCP 45
p <- NULL
for (i in 1:length(dfP.wk.45$year)){
  xi <- quantile(dfP.wk.45[i,2:14], 0.1)[[1]]
  p <- rbind(p,xi)
}
```

```
## Warning in xtfrm.data.frame(x): cannot xtfrm data frames

## Warning in xtfrm.data.frame(x): cannot xtfrm data frames

## Warning in xtfrm.data.frame(x): cannot xtfrm data frames

## Warning in xtfrm.data.frame(x): cannot xtfrm data frames

## Warning in xtfrm.data.frame(x): cannot xtfrm data frames

## Warning in xtfrm.data.frame(x): cannot xtfrm data frames

## Warning in xtfrm.data.frame(x): cannot xtfrm data frames

## Warning in xtfrm.data.frame(x): cannot xtfrm data frames

## Warning in xtfrm.data.frame(x): cannot xtfrm data frames

## Warning in xtfrm.data.frame(x): cannot xtfrm data frames

## Warning in xtfrm.data.frame(x): cannot xtfrm data frames

## Warning in xtfrm.data.frame(x): cannot xtfrm data frames

## Warning in xtfrm.data.frame(x): cannot xtfrm data frames

## Warning in xtfrm.data.frame(x): cannot xtfrm data frames

## Warning in xtfrm.data.frame(x): cannot xtfrm data frames

## Warning in xtfrm.data.frame(x): cannot xtfrm data frames

## Warning in xtfrm.data.frame(x): cannot xtfrm data frames

## Warning in xtfrm.data.frame(x): cannot xtfrm data frames

## Warning in xtfrm.data.frame(x): cannot xtfrm data frames

## Warning in xtfrm.data.frame(x): cannot xtfrm data frames

## Warning in xtfrm.data.frame(x): cannot xtfrm data frames

## Warning in xtfrm.data.frame(x): cannot xtfrm data frames

## Warning in xtfrm.data.frame(x): cannot xtfrm data frames

## Warning in xtfrm.data.frame(x): cannot xtfrm data frames

## Warning in xtfrm.data.frame(x): cannot xtfrm data frames

## Warning in xtfrm.data.frame(x): cannot xtfrm data frames

## Warning in xtfrm.data.frame(x): cannot xtfrm data frames

## Warning in xtfrm.data.frame(x): cannot xtfrm data frames

## Warning in xtfrm.data.frame(x): cannot xtfrm data frames

## Warning in xtfrm.data.frame(x): cannot xtfrm data frames

## Warning in xtfrm.data.frame(x): cannot xtfrm data frames

## Warning in xtfrm.data.frame(x): cannot xtfrm data frames

## Warning in xtfrm.data.frame(x): cannot xtfrm data frames

## Warning in xtfrm.data.frame(x): cannot xtfrm data frames

## Warning in xtfrm.data.frame(x): cannot xtfrm data frames

## Warning in xtfrm.data.frame(x): cannot xtfrm data frames

## Warning in xtfrm.data.frame(x): cannot xtfrm data frames

## Warning in xtfrm.data.frame(x): cannot xtfrm data frames

## Warning in xtfrm.data.frame(x): cannot xtfrm data frames

## Warning in xtfrm.data.frame(x): cannot xtfrm data frames

## Warning in xtfrm.data.frame(x): cannot xtfrm data frames

## Warning in xtfrm.data.frame(x): cannot xtfrm data frames

## Warning in xtfrm.data.frame(x): cannot xtfrm data frames

## Warning in xtfrm.data.frame(x): cannot xtfrm data frames

## Warning in xtfrm.data.frame(x): cannot xtfrm data frames

## Warning in xtfrm.data.frame(x): cannot xtfrm data frames

## Warning in xtfrm.data.frame(x): cannot xtfrm data frames

## Warning in xtfrm.data.frame(x): cannot xtfrm data frames

## Warning in xtfrm.data.frame(x): cannot xtfrm data frames

## Warning in xtfrm.data.frame(x): cannot xtfrm data frames

## Warning in xtfrm.data.frame(x): cannot xtfrm data frames

## Warning in xtfrm.data.frame(x): cannot xtfrm data frames

## Warning in xtfrm.data.frame(x): cannot xtfrm data frames

## Warning in xtfrm.data.frame(x): cannot xtfrm data frames

## Warning in xtfrm.data.frame(x): cannot xtfrm data frames

## Warning in xtfrm.data.frame(x): cannot xtfrm data frames

## Warning in xtfrm.data.frame(x): cannot xtfrm data frames

## Warning in xtfrm.data.frame(x): cannot xtfrm data frames

## Warning in xtfrm.data.frame(x): cannot xtfrm data frames

## Warning in xtfrm.data.frame(x): cannot xtfrm data frames

## Warning in xtfrm.data.frame(x): cannot xtfrm data frames

## Warning in xtfrm.data.frame(x): cannot xtfrm data frames

## Warning in xtfrm.data.frame(x): cannot xtfrm data frames

## Warning in xtfrm.data.frame(x): cannot xtfrm data frames

## Warning in xtfrm.data.frame(x): cannot xtfrm data frames

## Warning in xtfrm.data.frame(x): cannot xtfrm data frames

## Warning in xtfrm.data.frame(x): cannot xtfrm data frames

## Warning in xtfrm.data.frame(x): cannot xtfrm data frames

## Warning in xtfrm.data.frame(x): cannot xtfrm data frames

## Warning in xtfrm.data.frame(x): cannot xtfrm data frames

## Warning in xtfrm.data.frame(x): cannot xtfrm data frames

## Warning in xtfrm.data.frame(x): cannot xtfrm data frames

## Warning in xtfrm.data.frame(x): cannot xtfrm data frames

## Warning in xtfrm.data.frame(x): cannot xtfrm data frames

## Warning in xtfrm.data.frame(x): cannot xtfrm data frames

## Warning in xtfrm.data.frame(x): cannot xtfrm data frames

## Warning in xtfrm.data.frame(x): cannot xtfrm data frames

## Warning in xtfrm.data.frame(x): cannot xtfrm data frames

## Warning in xtfrm.data.frame(x): cannot xtfrm data frames

## Warning in xtfrm.data.frame(x): cannot xtfrm data frames

## Warning in xtfrm.data.frame(x): cannot xtfrm data frames
```

```
dfP.wk.45$p10 <- p

p <- NULL
for (i in 1:length(dfP.wk.45$year)){
  xi <- quantile(dfP.wk.45[i,2:14], 0.9)[[1]]
  p <- rbind(p,xi)
}
```

```
## Warning in xtfrm.data.frame(x): cannot xtfrm data frames

## Warning in xtfrm.data.frame(x): cannot xtfrm data frames

## Warning in xtfrm.data.frame(x): cannot xtfrm data frames

## Warning in xtfrm.data.frame(x): cannot xtfrm data frames

## Warning in xtfrm.data.frame(x): cannot xtfrm data frames

## Warning in xtfrm.data.frame(x): cannot xtfrm data frames

## Warning in xtfrm.data.frame(x): cannot xtfrm data frames

## Warning in xtfrm.data.frame(x): cannot xtfrm data frames

## Warning in xtfrm.data.frame(x): cannot xtfrm data frames

## Warning in xtfrm.data.frame(x): cannot xtfrm data frames

## Warning in xtfrm.data.frame(x): cannot xtfrm data frames

## Warning in xtfrm.data.frame(x): cannot xtfrm data frames

## Warning in xtfrm.data.frame(x): cannot xtfrm data frames

## Warning in xtfrm.data.frame(x): cannot xtfrm data frames

## Warning in xtfrm.data.frame(x): cannot xtfrm data frames

## Warning in xtfrm.data.frame(x): cannot xtfrm data frames

## Warning in xtfrm.data.frame(x): cannot xtfrm data frames

## Warning in xtfrm.data.frame(x): cannot xtfrm data frames

## Warning in xtfrm.data.frame(x): cannot xtfrm data frames

## Warning in xtfrm.data.frame(x): cannot xtfrm data frames

## Warning in xtfrm.data.frame(x): cannot xtfrm data frames

## Warning in xtfrm.data.frame(x): cannot xtfrm data frames

## Warning in xtfrm.data.frame(x): cannot xtfrm data frames

## Warning in xtfrm.data.frame(x): cannot xtfrm data frames

## Warning in xtfrm.data.frame(x): cannot xtfrm data frames

## Warning in xtfrm.data.frame(x): cannot xtfrm data frames

## Warning in xtfrm.data.frame(x): cannot xtfrm data frames

## Warning in xtfrm.data.frame(x): cannot xtfrm data frames

## Warning in xtfrm.data.frame(x): cannot xtfrm data frames

## Warning in xtfrm.data.frame(x): cannot xtfrm data frames

## Warning in xtfrm.data.frame(x): cannot xtfrm data frames

## Warning in xtfrm.data.frame(x): cannot xtfrm data frames

## Warning in xtfrm.data.frame(x): cannot xtfrm data frames

## Warning in xtfrm.data.frame(x): cannot xtfrm data frames

## Warning in xtfrm.data.frame(x): cannot xtfrm data frames

## Warning in xtfrm.data.frame(x): cannot xtfrm data frames

## Warning in xtfrm.data.frame(x): cannot xtfrm data frames

## Warning in xtfrm.data.frame(x): cannot xtfrm data frames

## Warning in xtfrm.data.frame(x): cannot xtfrm data frames

## Warning in xtfrm.data.frame(x): cannot xtfrm data frames

## Warning in xtfrm.data.frame(x): cannot xtfrm data frames

## Warning in xtfrm.data.frame(x): cannot xtfrm data frames

## Warning in xtfrm.data.frame(x): cannot xtfrm data frames

## Warning in xtfrm.data.frame(x): cannot xtfrm data frames

## Warning in xtfrm.data.frame(x): cannot xtfrm data frames

## Warning in xtfrm.data.frame(x): cannot xtfrm data frames

## Warning in xtfrm.data.frame(x): cannot xtfrm data frames

## Warning in xtfrm.data.frame(x): cannot xtfrm data frames

## Warning in xtfrm.data.frame(x): cannot xtfrm data frames

## Warning in xtfrm.data.frame(x): cannot xtfrm data frames

## Warning in xtfrm.data.frame(x): cannot xtfrm data frames

## Warning in xtfrm.data.frame(x): cannot xtfrm data frames

## Warning in xtfrm.data.frame(x): cannot xtfrm data frames

## Warning in xtfrm.data.frame(x): cannot xtfrm data frames

## Warning in xtfrm.data.frame(x): cannot xtfrm data frames

## Warning in xtfrm.data.frame(x): cannot xtfrm data frames

## Warning in xtfrm.data.frame(x): cannot xtfrm data frames

## Warning in xtfrm.data.frame(x): cannot xtfrm data frames

## Warning in xtfrm.data.frame(x): cannot xtfrm data frames

## Warning in xtfrm.data.frame(x): cannot xtfrm data frames

## Warning in xtfrm.data.frame(x): cannot xtfrm data frames

## Warning in xtfrm.data.frame(x): cannot xtfrm data frames

## Warning in xtfrm.data.frame(x): cannot xtfrm data frames

## Warning in xtfrm.data.frame(x): cannot xtfrm data frames

## Warning in xtfrm.data.frame(x): cannot xtfrm data frames

## Warning in xtfrm.data.frame(x): cannot xtfrm data frames

## Warning in xtfrm.data.frame(x): cannot xtfrm data frames

## Warning in xtfrm.data.frame(x): cannot xtfrm data frames

## Warning in xtfrm.data.frame(x): cannot xtfrm data frames

## Warning in xtfrm.data.frame(x): cannot xtfrm data frames

## Warning in xtfrm.data.frame(x): cannot xtfrm data frames

## Warning in xtfrm.data.frame(x): cannot xtfrm data frames

## Warning in xtfrm.data.frame(x): cannot xtfrm data frames

## Warning in xtfrm.data.frame(x): cannot xtfrm data frames

## Warning in xtfrm.data.frame(x): cannot xtfrm data frames

## Warning in xtfrm.data.frame(x): cannot xtfrm data frames

## Warning in xtfrm.data.frame(x): cannot xtfrm data frames

## Warning in xtfrm.data.frame(x): cannot xtfrm data frames

## Warning in xtfrm.data.frame(x): cannot xtfrm data frames

## Warning in xtfrm.data.frame(x): cannot xtfrm data frames

## Warning in xtfrm.data.frame(x): cannot xtfrm data frames
```

```
dfP.wk.45$p90 <- p

#RCP 85
p <- NULL
for (i in 1:length(dfP.wk.85$year)){
  xi <- quantile(dfP.wk.85[i,2:14], 0.1)[[1]]
  p <- rbind(p,xi)
}
```

```
## Warning in xtfrm.data.frame(x): cannot xtfrm data frames

## Warning in xtfrm.data.frame(x): cannot xtfrm data frames

## Warning in xtfrm.data.frame(x): cannot xtfrm data frames

## Warning in xtfrm.data.frame(x): cannot xtfrm data frames

## Warning in xtfrm.data.frame(x): cannot xtfrm data frames

## Warning in xtfrm.data.frame(x): cannot xtfrm data frames

## Warning in xtfrm.data.frame(x): cannot xtfrm data frames

## Warning in xtfrm.data.frame(x): cannot xtfrm data frames

## Warning in xtfrm.data.frame(x): cannot xtfrm data frames

## Warning in xtfrm.data.frame(x): cannot xtfrm data frames

## Warning in xtfrm.data.frame(x): cannot xtfrm data frames

## Warning in xtfrm.data.frame(x): cannot xtfrm data frames

## Warning in xtfrm.data.frame(x): cannot xtfrm data frames

## Warning in xtfrm.data.frame(x): cannot xtfrm data frames

## Warning in xtfrm.data.frame(x): cannot xtfrm data frames

## Warning in xtfrm.data.frame(x): cannot xtfrm data frames

## Warning in xtfrm.data.frame(x): cannot xtfrm data frames

## Warning in xtfrm.data.frame(x): cannot xtfrm data frames

## Warning in xtfrm.data.frame(x): cannot xtfrm data frames

## Warning in xtfrm.data.frame(x): cannot xtfrm data frames

## Warning in xtfrm.data.frame(x): cannot xtfrm data frames

## Warning in xtfrm.data.frame(x): cannot xtfrm data frames

## Warning in xtfrm.data.frame(x): cannot xtfrm data frames

## Warning in xtfrm.data.frame(x): cannot xtfrm data frames

## Warning in xtfrm.data.frame(x): cannot xtfrm data frames

## Warning in xtfrm.data.frame(x): cannot xtfrm data frames

## Warning in xtfrm.data.frame(x): cannot xtfrm data frames

## Warning in xtfrm.data.frame(x): cannot xtfrm data frames

## Warning in xtfrm.data.frame(x): cannot xtfrm data frames

## Warning in xtfrm.data.frame(x): cannot xtfrm data frames

## Warning in xtfrm.data.frame(x): cannot xtfrm data frames

## Warning in xtfrm.data.frame(x): cannot xtfrm data frames

## Warning in xtfrm.data.frame(x): cannot xtfrm data frames

## Warning in xtfrm.data.frame(x): cannot xtfrm data frames

## Warning in xtfrm.data.frame(x): cannot xtfrm data frames

## Warning in xtfrm.data.frame(x): cannot xtfrm data frames

## Warning in xtfrm.data.frame(x): cannot xtfrm data frames

## Warning in xtfrm.data.frame(x): cannot xtfrm data frames

## Warning in xtfrm.data.frame(x): cannot xtfrm data frames

## Warning in xtfrm.data.frame(x): cannot xtfrm data frames

## Warning in xtfrm.data.frame(x): cannot xtfrm data frames

## Warning in xtfrm.data.frame(x): cannot xtfrm data frames

## Warning in xtfrm.data.frame(x): cannot xtfrm data frames

## Warning in xtfrm.data.frame(x): cannot xtfrm data frames

## Warning in xtfrm.data.frame(x): cannot xtfrm data frames

## Warning in xtfrm.data.frame(x): cannot xtfrm data frames

## Warning in xtfrm.data.frame(x): cannot xtfrm data frames

## Warning in xtfrm.data.frame(x): cannot xtfrm data frames

## Warning in xtfrm.data.frame(x): cannot xtfrm data frames

## Warning in xtfrm.data.frame(x): cannot xtfrm data frames

## Warning in xtfrm.data.frame(x): cannot xtfrm data frames

## Warning in xtfrm.data.frame(x): cannot xtfrm data frames

## Warning in xtfrm.data.frame(x): cannot xtfrm data frames

## Warning in xtfrm.data.frame(x): cannot xtfrm data frames

## Warning in xtfrm.data.frame(x): cannot xtfrm data frames

## Warning in xtfrm.data.frame(x): cannot xtfrm data frames

## Warning in xtfrm.data.frame(x): cannot xtfrm data frames

## Warning in xtfrm.data.frame(x): cannot xtfrm data frames

## Warning in xtfrm.data.frame(x): cannot xtfrm data frames

## Warning in xtfrm.data.frame(x): cannot xtfrm data frames

## Warning in xtfrm.data.frame(x): cannot xtfrm data frames

## Warning in xtfrm.data.frame(x): cannot xtfrm data frames

## Warning in xtfrm.data.frame(x): cannot xtfrm data frames

## Warning in xtfrm.data.frame(x): cannot xtfrm data frames

## Warning in xtfrm.data.frame(x): cannot xtfrm data frames

## Warning in xtfrm.data.frame(x): cannot xtfrm data frames

## Warning in xtfrm.data.frame(x): cannot xtfrm data frames

## Warning in xtfrm.data.frame(x): cannot xtfrm data frames

## Warning in xtfrm.data.frame(x): cannot xtfrm data frames

## Warning in xtfrm.data.frame(x): cannot xtfrm data frames

## Warning in xtfrm.data.frame(x): cannot xtfrm data frames

## Warning in xtfrm.data.frame(x): cannot xtfrm data frames

## Warning in xtfrm.data.frame(x): cannot xtfrm data frames

## Warning in xtfrm.data.frame(x): cannot xtfrm data frames

## Warning in xtfrm.data.frame(x): cannot xtfrm data frames

## Warning in xtfrm.data.frame(x): cannot xtfrm data frames

## Warning in xtfrm.data.frame(x): cannot xtfrm data frames

## Warning in xtfrm.data.frame(x): cannot xtfrm data frames

## Warning in xtfrm.data.frame(x): cannot xtfrm data frames

## Warning in xtfrm.data.frame(x): cannot xtfrm data frames

## Warning in xtfrm.data.frame(x): cannot xtfrm data frames
```

```
dfP.wk.85$p10 <- p

p <- NULL
for (i in 1:length(dfP.wk.85$year)){
  xi <- quantile(dfP.wk.85[i,2:14], 0.9)[[1]]
  p <- rbind(p,xi)
}
```

```
## Warning in xtfrm.data.frame(x): cannot xtfrm data frames

## Warning in xtfrm.data.frame(x): cannot xtfrm data frames

## Warning in xtfrm.data.frame(x): cannot xtfrm data frames

## Warning in xtfrm.data.frame(x): cannot xtfrm data frames

## Warning in xtfrm.data.frame(x): cannot xtfrm data frames

## Warning in xtfrm.data.frame(x): cannot xtfrm data frames

## Warning in xtfrm.data.frame(x): cannot xtfrm data frames

## Warning in xtfrm.data.frame(x): cannot xtfrm data frames

## Warning in xtfrm.data.frame(x): cannot xtfrm data frames

## Warning in xtfrm.data.frame(x): cannot xtfrm data frames

## Warning in xtfrm.data.frame(x): cannot xtfrm data frames

## Warning in xtfrm.data.frame(x): cannot xtfrm data frames

## Warning in xtfrm.data.frame(x): cannot xtfrm data frames

## Warning in xtfrm.data.frame(x): cannot xtfrm data frames

## Warning in xtfrm.data.frame(x): cannot xtfrm data frames

## Warning in xtfrm.data.frame(x): cannot xtfrm data frames

## Warning in xtfrm.data.frame(x): cannot xtfrm data frames

## Warning in xtfrm.data.frame(x): cannot xtfrm data frames

## Warning in xtfrm.data.frame(x): cannot xtfrm data frames

## Warning in xtfrm.data.frame(x): cannot xtfrm data frames

## Warning in xtfrm.data.frame(x): cannot xtfrm data frames

## Warning in xtfrm.data.frame(x): cannot xtfrm data frames

## Warning in xtfrm.data.frame(x): cannot xtfrm data frames

## Warning in xtfrm.data.frame(x): cannot xtfrm data frames

## Warning in xtfrm.data.frame(x): cannot xtfrm data frames

## Warning in xtfrm.data.frame(x): cannot xtfrm data frames

## Warning in xtfrm.data.frame(x): cannot xtfrm data frames

## Warning in xtfrm.data.frame(x): cannot xtfrm data frames

## Warning in xtfrm.data.frame(x): cannot xtfrm data frames

## Warning in xtfrm.data.frame(x): cannot xtfrm data frames

## Warning in xtfrm.data.frame(x): cannot xtfrm data frames

## Warning in xtfrm.data.frame(x): cannot xtfrm data frames

## Warning in xtfrm.data.frame(x): cannot xtfrm data frames

## Warning in xtfrm.data.frame(x): cannot xtfrm data frames

## Warning in xtfrm.data.frame(x): cannot xtfrm data frames

## Warning in xtfrm.data.frame(x): cannot xtfrm data frames

## Warning in xtfrm.data.frame(x): cannot xtfrm data frames

## Warning in xtfrm.data.frame(x): cannot xtfrm data frames

## Warning in xtfrm.data.frame(x): cannot xtfrm data frames

## Warning in xtfrm.data.frame(x): cannot xtfrm data frames

## Warning in xtfrm.data.frame(x): cannot xtfrm data frames

## Warning in xtfrm.data.frame(x): cannot xtfrm data frames

## Warning in xtfrm.data.frame(x): cannot xtfrm data frames

## Warning in xtfrm.data.frame(x): cannot xtfrm data frames

## Warning in xtfrm.data.frame(x): cannot xtfrm data frames

## Warning in xtfrm.data.frame(x): cannot xtfrm data frames

## Warning in xtfrm.data.frame(x): cannot xtfrm data frames

## Warning in xtfrm.data.frame(x): cannot xtfrm data frames

## Warning in xtfrm.data.frame(x): cannot xtfrm data frames

## Warning in xtfrm.data.frame(x): cannot xtfrm data frames

## Warning in xtfrm.data.frame(x): cannot xtfrm data frames

## Warning in xtfrm.data.frame(x): cannot xtfrm data frames

## Warning in xtfrm.data.frame(x): cannot xtfrm data frames

## Warning in xtfrm.data.frame(x): cannot xtfrm data frames

## Warning in xtfrm.data.frame(x): cannot xtfrm data frames

## Warning in xtfrm.data.frame(x): cannot xtfrm data frames

## Warning in xtfrm.data.frame(x): cannot xtfrm data frames

## Warning in xtfrm.data.frame(x): cannot xtfrm data frames

## Warning in xtfrm.data.frame(x): cannot xtfrm data frames

## Warning in xtfrm.data.frame(x): cannot xtfrm data frames

## Warning in xtfrm.data.frame(x): cannot xtfrm data frames

## Warning in xtfrm.data.frame(x): cannot xtfrm data frames

## Warning in xtfrm.data.frame(x): cannot xtfrm data frames

## Warning in xtfrm.data.frame(x): cannot xtfrm data frames

## Warning in xtfrm.data.frame(x): cannot xtfrm data frames

## Warning in xtfrm.data.frame(x): cannot xtfrm data frames

## Warning in xtfrm.data.frame(x): cannot xtfrm data frames

## Warning in xtfrm.data.frame(x): cannot xtfrm data frames

## Warning in xtfrm.data.frame(x): cannot xtfrm data frames

## Warning in xtfrm.data.frame(x): cannot xtfrm data frames

## Warning in xtfrm.data.frame(x): cannot xtfrm data frames

## Warning in xtfrm.data.frame(x): cannot xtfrm data frames

## Warning in xtfrm.data.frame(x): cannot xtfrm data frames

## Warning in xtfrm.data.frame(x): cannot xtfrm data frames

## Warning in xtfrm.data.frame(x): cannot xtfrm data frames

## Warning in xtfrm.data.frame(x): cannot xtfrm data frames

## Warning in xtfrm.data.frame(x): cannot xtfrm data frames

## Warning in xtfrm.data.frame(x): cannot xtfrm data frames

## Warning in xtfrm.data.frame(x): cannot xtfrm data frames

## Warning in xtfrm.data.frame(x): cannot xtfrm data frames

## Warning in xtfrm.data.frame(x): cannot xtfrm data frames
```

```
dfP.wk.85$p90 <- p
```

Calculate rate models are increasing

```
#Identify intercept- Method 1: means of real data we have
yintercept= mean(dfB.wk$real.wk)
xinitial=mean(dfB.wk$year)

#Set up loop
Model_list <- unique(dfP$GCM) #List to loop through
L <- length(Model_list)
coeff.table <- NULL #Empty table to hold results

#loop through all the models
for (i in 1:L){
  model_i <- Model_list[i] #specify which model
  df.sub <- subset(dfP.wk, dfP.wk$GCM == model_i) #subset df of all model avgs to model of interest
  df.sub <- subset(df.sub, year > 2019)
  
  #Linear transformation of year and yint so that line forced through origin (0,0)
  df.sub$year <- df.sub$year-xinitial
  df.sub$pred.wk <- df.sub$pred.wk - yintercept
  
  #Linear regression forced through origin
  lm <- summary(lm(formula=pred.wk ~ year + 0, data=df.sub)) 
  coeff <- c(model_i, lm$coefficients[[1]], lm$coefficients[[2]], lm$coefficients[[4]] ) #Extract useful data
  coeff.table <- rbind(coeff.table, coeff) #bind onto dataframe
}

#Format coefficient table
colnames(coeff.table) <- c("model", "year_est", "year_std_error", "year_p")
coeff.table <- as.data.frame(coeff.table)
coeff.table$year_std_error <- as.numeric(coeff.table$year_std_error)
coeff.table$year_est <- as.numeric(coeff.table$year_est)

#Calculate CI
coeff.table$CI.low <- coeff.table$year_est - 1.96*coeff.table$year_std_error
coeff.table$CI.high <- coeff.table$year_est + 1.96*coeff.table$year_std_error

#Add RCP labels for plotting
coeff.table$RCP <- str_sub(coeff.table$model, -2, -1)
coeff.table$GCM <- str_sub(coeff.table$model, end = -7)

df.lm45 <- subset(coeff.table, coeff.table$RCP=="45")
df.lm45 <- data.frame(df.lm45$GCM, df.lm45$year_est, df.lm45$CI.low, df.lm45$CI.high)
colnames(df.lm45) <- c("GCM", "Est_45", "CIlow_45", "CIhigh_45")
df.lm85 <- subset(coeff.table, coeff.table$RCP=="85")
df.lm85 <- data.frame(df.lm85$GCM, df.lm85$year_est, df.lm85$CI.low, df.lm85$CI.high)
colnames(df.lm85) <- c("GCM", "Est_85", "CIlow_85", "CIhigh_85")

df.coeff <- merge(df.lm45, df.lm85, by="GCM")
df.coeff
```

```
##               GCM      Est_45    CIlow_45   CIhigh_45      Est_85    CIlow_85
## 1         CanESM2 0.003538903 0.002320329 0.004757476 0.010815375 0.008575013
## 2        CNRM-CM5 0.006349207 0.004395694 0.008302720 0.010748804 0.008688857
## 3   CSIRO-Mk3-6-0 0.011017142 0.008563620 0.013470664 0.012368749 0.010646709
## 4      GFDL-ESM2G 0.010099855 0.007943809 0.012255901 0.015250197 0.013043522
## 5      GFDL-ESM2M 0.005972358 0.003819523 0.008125192 0.010392260 0.008314534
## 6   HadGEM2-CC365 0.008916750 0.007261846 0.010571653 0.015975204 0.014015506
## 7   HadGEM2-ES365 0.008805543 0.007278304 0.010332781 0.015130230 0.013523715
## 8          inmcm4 0.003809500 0.001928763 0.005690236 0.012014170 0.010395601
## 9    IPSL-CM5A-LR 0.007802826 0.006034687 0.009570965 0.015435019 0.013681680
## 10   IPSL-CM5A-MR 0.006361603 0.004915920 0.007807285 0.012462477 0.010870986
## 11      MIROC-ESM 0.009834576 0.007986450 0.011682702 0.024629320 0.022093174
## 12 MIROC-ESM-CHEM 0.012610449 0.010452500 0.014768398 0.032091661 0.029963238
## 13         MIROC5 0.008001680 0.006147033 0.009856327 0.011905522 0.010252804
## 14      MRI-CGCM3 0.004671214 0.001263005 0.008079423 0.009594737 0.007881607
##     CIhigh_85
## 1  0.01305574
## 2  0.01280875
## 3  0.01409079
## 4  0.01745687
## 5  0.01246999
## 6  0.01793490
## 7  0.01673674
## 8  0.01363274
## 9  0.01718836
## 10 0.01405397
## 11 0.02716547
## 12 0.03422008
## 13 0.01355824
## 14 0.01130787
```

# Figure 1

Projected HRI

```
fig1a <- ggplot()+
  theme_classic()+
  geom_line(data=df.grid.wk, aes(x=year, y=pred.wk), size=2)+ #historical pred
  geom_line(data=dfP.wk%>%filter(RCP=="45"), aes(x=year, y=pred.wk, group=GCM),color= "lightblue3", size= 1.75, alpha=0.5)+ #RCP 45
  geom_errorbar(data=dfB.wk, aes(x=year, ymin=real.LB, ymax=real.UB), colour="slateblue1", size=1, alpha=0.5)+  #Error bars
  geom_point(data=dfB.wk, aes(x=year, y=real.wk), shape = 21, colour = "black", fill = "slateblue1", size=3)+ #real data
  geom_line(data=dfP.wk.45, aes(x=year, y=p10),linetype = "twodash", size=1)+ #10% line
  geom_line(data=dfP.wk.45,  aes(x=year, y=p90),linetype = "twodash", size=1)+ #90% line
  scale_x_continuous(breaks = seq(1980, 2100, 20), lim = c(1979, 2100))+ #Scale the x axis
  scale_y_continuous(breaks = seq(0, floor(max(dfP.wk$pred.wk))+1, 1), 
                     lim = c(0, floor(max(dfP.wk$pred.wk))+1), expand = c(0, 0))+
  xlab("Year")+ #X axis label
  ylab("Avg. weekly HRI per season per 100k visitors")+ #Y axis label
  theme(axis.text.x = element_text(size=12, angle=-45, vjust=0.7, hjust=0.1),
        axis.text.y = element_text(size=12),
        plot.title = element_text(hjust = 0.5),
        axis.title.x = element_text(size=14),
        axis.title.y = element_text(size=14))+
  ggtitle("RCP 4.5") #title
```

```
## Warning: Using `size` aesthetic for lines was deprecated in ggplot2 3.4.0.
## ℹ Please use `linewidth` instead.
```

```
fig1b <- ggplot()+
  theme_classic()+
  geom_line(data=df.grid.wk, aes(x=year, y=pred.wk), size=2)+ #historical pred
  geom_line(data=dfP.wk%>%filter(RCP=="85"), aes(x=year, y=pred.wk, group=GCM),color= "salmon", size= 1.75, alpha=0.5)+ #RCP 45
  geom_errorbar(data=dfB.wk, aes(x=year, ymin=real.LB, ymax=real.UB), colour="slateblue1", size=1, alpha=0.5)+  #Error bars
  geom_point(data=dfB.wk, aes(x=year, y=real.wk), shape = 21, colour = "black", fill = "slateblue1", size=3)+ #real data
  geom_line(data=dfP.wk.85, aes(x=year, y=p10),linetype = "twodash", size=1)+ #10% line
  geom_line(data=dfP.wk.85,  aes(x=year, y=p90),linetype = "twodash", size=1)+ #90% line
  scale_x_continuous(breaks = seq(1980, 2100, 20), lim = c(1979, 2100))+ #Scale the x axis
  scale_y_continuous(breaks = seq(0, floor(max(dfP.wk$pred.wk))+1, 1), lim = c(0, floor(max(dfP.wk$pred.wk))+1), expand = c(0, 0))+
  xlab("Year")+ #X axis label
  ylab("Avg. weekly HRI per season per 100k visitors")+ #Y axis label
  theme(axis.text.x = element_text(size=12, angle=-45, vjust=0.7, hjust=0.1),
        axis.text.y = element_text(size=12),
        plot.title = element_text(hjust = 0.5),
        axis.title.x = element_text(size=14),
        axis.title.y = element_text(size=14))+
  ggtitle("RCP 8.5") #title

fig1a
```

```
fig1b
```

# Figure 2

Amt of increase per model

```
fig2 <- ggplot()+
  theme_classic()+
  geom_errorbar(data=df.coeff, aes(xmin = CIlow_45, xmax= CIhigh_45, y=reorder(GCM, -Est_45), color= "RCP 4.5"), width=0.5, size=1) + #4.5 error bars
  geom_point(df.coeff, mapping= aes(x= Est_45, y=reorder(GCM, -Est_45), color="RCP 4.5", shape="RCP 4.5"), size =3)+ #4.5 estimates
  geom_errorbar(data=df.coeff, aes(xmin = CIlow_85, xmax= CIhigh_85, y=GCM, color= "RCP 8.5"), width=0.5, size=1) + #8.5 error bars
  geom_point(df.coeff, mapping= aes(x= Est_85, y=GCM, color="RCP 8.5", shape="RCP 8.5"), size=3)+ #4.5 estimates
  scale_color_manual(values=c(
    "RCP 4.5" = "lightblue3", 
    "RCP 8.5" = "salmon"),
    name= "Legend")+
  scale_shape_manual(values=c(
    "RCP 4.5" = 15, 
    "RCP 8.5" = 19),
    name= "Legend")+
  xlab("Increase in heat events per 100k visitors per year")+
  ylab("Global climate model")+
  ggtitle("Projected increase in mean weekly heat events")
fig2
```
