# Supplementary material for: Predicting climate-change induced heat-related illness risk in Grand Canyon National Park visitors
Source: PLoS One. 2023 Aug 9;18(8):e0288812. doi: 10.1371/journal.pone.0288812 (PMC10411749; doi:10.1371/journal.pone.0288812)
Supplement: Attachment — Submitted filename: Climate Change.pdf [file pone.0288812.s007.pdf]

Climate change will increase heat-related illness risk in Grand Canyon National Park visitors

Section		Comment
General		**The article is well-written. The arguments are supported with references. However, some modifications are recommended to enhance the readability of the paper. Overall, consider revising the language in the manuscript for minor errors and the reference style, and add more figures. The resolution of the figures needs to be enhanced with at least 300 dpi.**
Title<br><br>Keywords		• The authors already mentioned in the *Introduction* that climate change increases HRI risk. You may consider a more specific title for the investigation.<br>• Remove the first two keywords since they are redundant with the title. Add some more descriptive keywords.
1.	Introduction	• Rewrite the following sentences for clarity and check the grammar.<br>- Line 48: "In addition to…"<br>- Line 73: "This trend…"<br>- Line 98: "This information…"<br>• The introduction provides a good background of the issue. However, it lacks studies from the literature concerning the topic.
2.	Materials and Methods	• Check the grammar for the first sentence and try to shorten it.<br>• In line 107: "GCNP saw a peak…". The word "saw" seems non-scientific. You may rewrite the sentence as follows: "According to ____, …"<br>• In line 117: Is it paper or papers?<br>• In line 119, rewrite the sentence. In line 123: "To be a case…", rewrite. In line 124: indicator or indicators?<br>• I recommend presenting the data in lines 136-139 in a table and commenting on the main observations only in the main text.<br>• Revise the sentence in line 145. Also, introduce the meanings of T_{min} and T_{max} if you mention them for the first time.<br>• Edit these sentences as follows: "World Climate Research Programme's Coupled Model Intercomparison Project phase 5 (CMIP5) multi-model dataset (Taylor et al., 2012), statistically downscaled using the Multivariate Adaptive Constructed Analogs (MACA)<br>• Break the sentence in line 165 after the word "projections".<br>• Please provide one or more figures in the "*Data*" sub-section to briefly describe the method.<br>• In lines 184-188, what do you mean by binned? "Week was assigned as Sunday-Monday. What do you mean? Rewrite the last sentence for clarity.<br>• What does SI stand for in line 194?

Section		Comment
		• In line 200, isn't Figure 1 a part of the results? Why is it presented in the methods? (The term 'projection' refers to a climate model [e.g., HadGEM2-CC365] driven by a specific radiative forcing [e.g., RCP 4.5] Figure 2, Supplemental Table 2). ??? Where are Supplemental Tables? And why is Table 2 cited before Table 1?
**3.**	**Results**	• How do you define the "summer" (i.e., time period) and "events" in your study.<br>• Is there a figure to highlight the main results (peak, min., avg., etc…). Is is Figure 1? If yes, why is it not cited in the "*Results*".<br>• In Table 1, add a space in "Interaction of RH_{min}".<br>• The captions of the figures are too long. Consider shortening them, and write the explanations in the main text when you describe the figures.
**5.**	**Discussion**	• Where is the discussion of the metrics in Table 1?<br>• Provide references for the sentence ending in line 292.<br>• Rewrite the sentence in line 277.<br>• Provide references whenever possible to the discussed points.
**6.**	**Conclusions**	The conclusions are too general. Try providing specific significant conclusions, and give recommendations for future work.
