# Supplementary material for: Predicting climate-change induced heat-related illness risk in Grand Canyon National Park visitors
Source: PLoS One. 2023 Aug 9;18(8):e0288812. doi: 10.1371/journal.pone.0288812 (PMC10411749; doi:10.1371/journal.pone.0288812)
Supplement: Attachment — Submitted filename: Response to reviewers comments.docx [file pone.0288812.s008.docx]

Dear Members of the Editorial Board,

We are pleased to resubmit for your consideration our manuscript now entitled, “Predicting climate-change induced heat-related illness in Grand Canyon National Park visitors to improve prevention” **(PONE-D-22-35008)**.

This resubmission is accompanied by thorough and specific responses in blue to the reviewers' comments and concerns. We thank the reviewers for their rigorous reading and helpful critique of our manuscript – addressing their comments and concerns has improved the scientific quality and readability of our manuscript.

In its current form, we expect this manuscript to enhance the body of knowledge about the relationship between climate change and heat-related illness in Grand Canyon National Park – and the associated considerations needed to design mitigation strategies – and to be of particular interest to the readership of your journal.

We thank you very much for the continued consideration of our manuscript.

Sincerely,

Danielle Buttke, DVM, PhD, MPH *(on behalf of all co-authors)*

National Park Service Office of Public Health

**REVIEWER #1**

The study titled “Climate change will increase heat-related illness risk in Grand Canyon National Park visitors" seems an interesting study contributed by authors. However, there is more space for authors to make it more effective for the research community. Therefore, major suggestions regarding this study area as follows:

1. The title of the research paper may be changed.

Thank you for your suggestion. We have changed the title to be more specific. The updated title is as follows:

Predicting climate-change induced heat-related illness in Grand Canyon National Park visitors to improve prevention

2. Authors need to write a clear research gap in the introduction section of this research study.

We have attempted to address this oversight and modified the introduction to now include language regarding the utility of this model to allow data-driven public health response to HRI, including: ‘staffing for this program is limited to the peak visitation season due to expected visitor numbers, which may not mirror peak HRI risk. .... Additionally, most public lands lack these resources and do not have a data-informed model to appropriately target limited resources to best affect public health outcomes.’ and ‘This information will allow the development of adaptive SAR and education programming targeting the highest risk conditions to improve public health outcomes as well as improve public land managers' understanding of how climate change threatens their mission to provide for safe recreation experiences for the benefit of this and future generations.

Added to abstract: Excessive risk during the shoulder season months presents an opportunity to increase preventative search and rescue and education efforts to mitigate increased risk.

3. There are many grammatical mistakes in the paper. Those cannot be pointed out one by one. So, it is recommended that this paper should be reviewed from English expert for proof reading to make it more attractive.

Thank you for your close reading. We have had the paper additionally reviewed internally at the National Park Service to correct grammatical errors.

4. Why gridMET gridded data set was used in this study? You can also try to do this with other gridded datasets such as CRU etc. Furthermore, compare their results with gridMET gridded dataset.

We used the GridMET historical dataset (Abatzoglou 2013) because it is used to train the GCMs in the MACA downscaling process, allowing us to directly compare the historical data to projections without having to perform additional bias corrections (and introduce additional error to the calculations). At 1/24 degree resolution, the GridMET dataset is finer scale than the suggested CRU data, which is preferred for the analysis.

The GridMET historical dataset is regularly used for analysis in this region so there is no need to compare with other datasets, which would be beyond the scope of this study.

5. Boundaries of study area and total area of the study area may be identified using Google earth engine or ArcGIS in the revised section of the paper.

A map has been added to the manuscript as Figure 1, copied below

**Figure 1**: Map of study site – Grand Canyon National Park is depicted in white. Park boundary data are publicly available through NPS IRMA and base map data is publicly available from ESRI.

Additionally, the following text has been added to the methods.

“We made the map using the “leaflet” package and other figures using the “ggplot2” package [(Wickham et al., 2016)](https://www.zotero.org/google-docs/?ndG0C1). “

6. The authors can prepare a table including all type of collected data and GCMs used in the study.
A table, detailing the types of collected data and GCMs, has been added to the manuscript and copied below.

**Table 1:** Sources of data used in regression models.

| **Data type** | **Purpose** | **Source** |
| --- | --- | --- |
| HRI events | Regression outcome | Noe et al, 2013 |
| Visitation records | Regression predictor | GCNP visitor use statistics program |
| Historical climate data (recorded daily T_min_, T_max_, RH_min_, RH_max_) | Regression predictor | GridMET: gridded daily surface meteorological dataset (Abatzoglou, 2013) |
| Projected climate data  (predicted daily T_max_, RH_min_) from 14 GCMs (global climate models) | Prediction input | World Climate Research Programme's Coupled Model Intercomparison Project phase 5 (CMIP5) multi-model dataset (Taylor et al., 2012), statistically downscaled using the MACA (Multivariate Adaptive Constructed Analogs) method (Abatzoglou & Brown, 2012).” |

7. The data collection period of 2004-2009 for prediction of HRI is less period. However, it is suggested for the authors to collect more data from 2010 and onwards to make regression model more effective if the data is easily available for authors from literature review or from concerned NPS authorities.

We agree that the dates used in the model are not ideal given the lag in time between data collection and present day. However, in 2009, Grand Canyon National Park switched to an electronic data collection system from the paper forms used previously, making data after 2009 not directly comparable to data prior to the data format change. Additionally, the newer electronic medical record system does not incorporate all of the same data streams used in the Noe et al. Paper, and heat illness is not coded as a defined outcome variable, requiring medical chart review of the free text fields of each case in order to categorize a victim as heat illness. We are currently working to understand how historic, multiple-source paper and current electronic medical record systems differ in the data captured to allow a direct comparison of data streams in future years.

8. Why these 14 GCMs were used in your study? You can review some literature which indicates the best skillful GCMs for the study area and can be used in this study. By doing this, results would be more reliable and authentic.

Using climate models to identify potential vulnerabilities and robust adaptation strategies does not require accurate prediction of future climate, rather a broad range of plausible representations of future climate (Dessai et al. 2009). To characterize the broadest possible range, we started with the full set of twenty global climate models (GCMs) available for CONUS from the MACA dataset (footnote: [MACA Statistical Downscaling Method (northwestknowledge.net)](https://climate.northwestknowledge.net/MACA/GCMs.php)). To ensure plausibility, four models were eliminated due to limited skill in the Southwestern U.S. (bcc-csm1-1, bcc-csm1-1-m, BNU-ESM, IPSL-CM5B-LR) (Rupp et al. 2013, 2016a,b Joyce and Coulson 2020, Schuurman et al. 2020) and two additional models were excluded from the set (NorESM1-M and CCSM4) because they do not have Rh data available, which is needed to calculate heat index.

Dessai, S., M. Hulme, R. Lempert, and R. Pielke Jr. 2009. Do we need better predictions to adapt to a changing climate? Eos Transactions, American Geophysical Union 90:111–112.

Rupp, D. E., J. T. Abatzoglou, K. C. Hegewisch, and P. W. Mote. 2013. Evaluation of CMIP5 20th century climate simulations for the Pacific Northwest USA. Journal of Geophysical Research Atmospheres 118:10,884-10,906.

Rupp, D.E. 2016a. An evaluation of CMIP5 20th century climate for the Southeastern United States as simulated by Coupled Model Intercomparison Project Phase 5 (CMIP5) global climate models. Open-File Report 2016- 1047. Reston, VA: U.S. Geological Survey. 32 p. <https://doi.org/10.3133/ofr20161047> [Accessed September 17, 2019].

Rupp, D.E. 2016b. Figures from an unpublished report of an evaluation of CMIP 20th century climate for the Southwestern United States as simulated by Coupled Model Intercomparison Project Phase 5 global climate models. Obtained from David Rupp, Oregon State University, 2016.

Joyce, L. A., and D. Coulson. 2020. Climate Scenarios and Projections: A technical document supporting the usda forest service 2020 rpa assessment. USDA Forest Service - General Technical Report RMRS-GTR 2020:1–85.

Schuurman, G.W., A. Symstad, B.W. Miller, A.N. Runyon, and R. Ohms. 2019. Climate change scenario planning for resource stewardship: Applying a novel approach in Devils Tower National Monument. Natural Resource Report NPS/XXXX/NRR—2019/XXXX. National Park Service, Fort Collins, Colorado.

9. The results of Figure 1 are not clear. Furthermore, Figure 1 results can also be done at 99% CI band to explore more about observed heat events.
We have uploaded the figures as separate files into the PLOS system, as well as re-copied them into the manuscript for ease of review. Hopefully, this improves their clarity.

**Figure 1:** Modeled heat events per 100,000 visitors in Grand Canyon National Park from 1979 through 2100. A Poisson regression was constructed using observed heat events in GRCA from 2004-2009. Represented here is the daily average per each year of both the modeled (black line) and each year’s daily average observed events (purple points) and its 95% confidence interval (purple error bars). Additionally the regression model was applied to 14 global climate models and projected heat events are shown in blue (RCP 4.5) and red (RCP 8.5) for each model with the 10th and 90th percentile of the ensemble marked with a dotted line.

11. The conclusion section of this study does not reflect the outcomes of the whole study. Therefore, it should be revised to include major contributions.

Thank you for the recommendation. We have now expanded the conclusion section to reflect the broader outcomes of the study. Specifically, we have added ‘with out-of-season extreme heat increasing heat illness risk more than in-season extreme heat, as well as extreme heat occurring during low humidity’ and ‘Because RCP 8.5 climate models most accurately track historic and current warming trends, the highest heat illness risk projections are the most likely and make heat illness risk a significant need for public land management planning and staffing efforts.’

**REVIEWER #2**

**General**

The article is well-written. The arguments are supported with references. However, some modifications are recommended to enhance the readability of the paper. Overall, consider revising the language in the manuscript for minor errors and the reference style, and add more figures. The resolution of the figures needs to be enhanced with at least 300 dpi.

We thank you for your comments. We have added the recommendations modifications below and have uploaded the figures as separate files into the PLOS system, as well as re-copied them into the manuscript for ease of review. Hopefully, this improves their clarity.

**Title**

The authors already mentioned in the Introduction that climate change increases HRI risk. You may consider a more specific title for the investigation.

We have changed the title to now read ‘Predicting climate-change induced heat-related illness risk in Grand Canyon National Park visitors to improve prevention’

**Keywords**

Remove the first two keywords since they are redundant with the title. Add some more descriptive keywords.

As keywords are sometimes used for indexing purposes in literature reviews and searches, we prefer to keep these terms as keywords.

**Introduction**

Rewrite the following sentences for clarity and check the grammar:

- Line 48: “In addition to…”

Line 48 has been rewritten for clarity and copied below.

“Heat-related illness (HRI) includes acute conditions of heat cramps, heat exhaustion and heat stroke, but can also exacerbate underlying health conditions such as respiratory or heart disease, diabetes, or cerebrovascular disease (Schwartz et al., 2015; Stonehill, 1972; Schulte et al. 2021).”

- Line 73: “This trend…”

Line 73 has been rewritten for clarity and copied below.

“Unanticipated heat during historically cooler months and in park units with historically milder climates increases the risk of HRI for unprepared visitors.”

- Line 98: “This information…”

Line 98 has been rewritten for clarity and to address another reviewer’s comment and copied below.

“This information will allow the development of adaptive SAR and education programming targeting the highest risk conditions to improve public health outcomes as well as improve public land managers' understanding of how climate change threatens their mission to provide for safe recreation experiences for the benefit of this and future generations.”

The introduction provides a good background of the issue. However, it lacks studies from the literature concerning the topic.

Aside from the Malcolm et al. Paper and the associated 2014 abstract from a Wilderness Medicine Symposium with the same data presented, we have found few studies that address the specific issue of heat illness in National Park Service visitors. We have added the following reference to the introduction:

Pearce et al., 2019, and welcome suggestions from the reviewers.

**Materials and Methods**

Check the grammar for the first sentence and try to shorten it.

The line has been rewritten for clarity and copied below

“Our study site, GCNP, provides a unique opportunity to understand HRI in a national-park context because of its well documented history of individual HRI incidents and reliable information about visitor numbers.”

In line 107: “GCNP saw a peak…”. The word “saw” seems non-scientific. You may rewrite the sentence as follows: “According to ____, …”

The line has been rewritten for clarity and copied below

“In 2018, visitor numbers to GCNP reached a peak of 6.38 million people with most visits occurring during the summer months (NPS n.d.).”

In line 117: Is it paper or papers?

The singular 'paper' serves as the modifier for records. We have clarified the language in the manuscript and copied it below.

“Both paper and electronic medical records were reviewed....”

In line 119, rewrite the sentence.

We have clarified the sentence and copied it below.

“HRI events were defined as:”

In line 123: “To be a case…”, rewrite. In line 124: indicator or indicators?

This is a quote from the original manuscript publishing the HRI data (Noe et al, 2013) in which the case definition was defined. We feel it is most appropriate to leave the original quote block unchanged so as not to deceive readers of the source material.

I recommend presenting the data in lines 136-139 in a table and commenting on the main observations only in the main text.

We disagree with the suggestion. The text referred to simply explains that most (>75%) visitors arrive by automobile, and how the remaining share is distributed among bus-, train-, and boat-based travel. Providing this information in tabular form would require much more space (for the table and caption), as well as reference to it in the main text, and so the reviewer-proposed alternative seems like a less elegant approach.

Revise the sentence in line 145. Also, introduce the meanings of Tmin and Tmax if you mention them for the first time.

The line has been revised for clarity and copied below.

“Historical climate data, including daily minimum temperature (T_min_), maximum temperature (T_max_), minimum relative humidity (RH_min_), and maximum relative humidity (RH _max_), were extracted from the Abatzoglou gridded daily surface meteorological (gridMET) dataset, for the period from 1979 to 2018 (Abatzoglou, 2013).”

Edit these sentences as follows: “World Climate Research Programme's Coupled Model Intercomparison Project phase 5 (CMIP5) multi-model dataset (Taylor et al., 2012), statistically downscaled using the Multivariate Adaptive Constructed Analogs (MACA)

The line has been revised for clarity and copied below.

“Projected climate data, including daily T_max_ and RH_min_ estimates, were extracted from 14 global climate models (GCMs) in the World Climate Research Programme's Coupled Model Intercomparison Project phase 5 (CMIP5) multi-model dataset (Taylor et al., 2012), statistically downscaled using the MACA (Multivariate Adaptive Constructed Analogs) method (Abatzoglou & Brown, 2012).”

Break the sentence in line 165 after the word “projections”.

The new text is copied below.

“We worked with climate uncertainty by considering a range of plausible climate futures (Runyon et al., 2020; Lawrence et 164al., 2021) represented by 28 projections. These 28 projections consisted of two simulations each of 14 downscaled CMIP5 global climate models, in which one simulation used a moderate greenhouse gas emissions pathway that assumes lower future emissions rates due to technological advancements and policy change (Representative Concentration Pathway [RCP] 4.5) and the other used a high emissions pathway (RCP 8.5) (Moss et al., 2010).”

Please provide one or more figures in the “Data” sub-section to briefly describe the method.

Table 1, detailing the types of collected data and GCMs, has been added to the manuscript and copied below, as well as Figure 1 describing the study site. Both have been copied below.

**Table 1:** Sources of data used in regression models.

| **Data type** | **Purpose** | **Source** |
| --- | --- | --- |
| HRI events | Regression outcome | Noe et al, 2013 |
| Visitation records | Regression predictor | GCNP visitor use statistics program |
| Historical climate data (recorded daily T_min_, T_max_, RH_min_, RH_max_) | Regression predictor | GridMET: gridded daily surface meteorological dataset (Abatzoglou, 2013) |
| Projected climate data  (predicted daily T_max_, RH_min_) from 14 GCMs (global climate models) | Prediction input | World Climate Research Programme's Coupled Model Intercomparison Project phase 5 (CMIP5) multi-model dataset (Taylor et al., 2012), statistically downscaled using the MACA (Multivariate Adaptive Constructed Analogs) method (Abatzoglou & Brown, 2012).” |

In lines 184-188, what do you mean by binned? “Week was assigned as Sunday-Monday. What do you mean? Rewrite the last sentence for clarity.

Data binning is a data pre-processing method: wikipedia.org/wiki/Data_binning

We have edited the text for clarity:

“Therefore, we binned the data (structured by date) into weeks, with weeks starting on Sunday and ending on Saturday.”

What does SI stand for in line 194?

S1 stands for Supplement 1.

In line 200, isn’t Figure 1 a part of the results? Why is it presented in the methods? (The term ‘projection’ refers to a climate model [e.g., HadGEM2-CC365] driven by a specific radiative forcing [e.g., RCP 4.5] Figure 2, Supplemental Table 2). ??? Where are Supplemental Tables? And why is Table 2 cited before Table 1?

We have removed the reference to Figure 1 in line 200. We have updated our Table names and supplemental files and added the following captions at the end of the manuscript.

**S1:** Deidentified binned data of visitation estimates and recorded HRIs used in regressions.

**S2:** Table of AIC of different Poisson models tested

**S3:** Table of predicted mean increase in cases per year for each climate model, estimated using lm(Events ~ year). Events increased per year taken as coefficient of slope [95% CI].

**S4:** Figure of recorded heat events (black) and predicted heat events (red line) at Grand Canyon National Park from 2004-2009; the pink shaded areas representing 95% prediction confidence intervals from the final negative binomial regression model.

**S5:** R code of regression model fitting.

**S6:** R code of regression model prediction.

**Results**

How do you define the “summer” (i.e., time period) and “events” in your study.

These definitions have been clarified in the text. We have also replaced the term ‘summer’ with the term ‘peak season’ throughout the text to avoid confusion

Line 119 was updated to read:

“HRI events were defined as: ….”

Line 184:

“Our primary outcome was the number of HRIs per week per estimated 100,000 visitors during the peak season. The peak season here is defined as April 1 through September 30.”

Is there a figure to highlight the main results (peak, min., avg., etc…). Is is Figure 1? If yes, why is it not cited in the “Results”.

Figure 1 is cited in the second paragraph of the Results and further expanded upon in the third paragraph.

In Table 1, add a space in “Interaction of RHmin”.

Thank you, the change has been updated in the Table.

**Table 2:** Variables, model estimates, standard error, and p-values of parameters evaluated for inclusion in the final regression model predicting the rate of heat related illness events in Grand Canyon National Park visitors, May through October, 2004-2009.

| **Variable** | **Estimate** | **Standard error** | ***p*-value** |
| --- | --- | --- | --- |
| Intercept | -11.85 | 1.21 | <0.01 |
| Average weekly maximum temperature (T_max_) | 0.03 | 0.01 | 0.04 |
| Average weekly minimum relative humidity (RH_min_) | -0.1 | 0.05 | 0.04 |
| May (compared to April reference) | 0.09 | 0.75 | 0.91 |
| June (compared to April reference) | -0.28 | 0.8 | 0.72 |
| July (compared to April reference) | -0.27 | 0.77 | 0.73 |
| August (compared to April reference) | -0.88 | 0.81 | 0.28 |
| September (compared to April reference) | -2.3 | 0.92 | 0.01 |
| Interaction of RH_min_ and May | 0.06 | 0.05 | 0.28 |
| Interaction of RH_min_ and June | 0.04 | 0.06 | 0.54 |
| Interaction of RH_min_ and July | 0.04 | 0.05 | 0.4 |
| Interaction of RH_min_ and August | 0.08 | 0.05 | 0.15 |
| Interaction of RH_min_ and September | 0.17 | 0.06 | <0.01 |

The captions of the figures are too long. Consider shortening them, and write the explanations in the main text when you describe the figures.

Journal guidelines for authors require figures to be stand-alone items, which require adequate description in the figure caption to describe the figure in its entirety without reference to the main text. Therefore, we have left the figure captions as-is.

**Discussion**

Where is the discussion of the metrics in Table 1?

These metrics are discussed in the first paragraph of the discussion.

Provide references for the sentence ending in line 292.

We have added a reference accordingly.

Rewrite the sentence in line 277.

The new text reads:

“These results suggest the RCP 8.5 models may more closely predict the actual increase in HRI most likely to be seen at GCNP and predict a concerning increase in disease burden among visitors.”

Provide references whenever possible to the discussed points.

Thank you for the suggestion, we have attempted to do so.

**Conclusions**

The conclusions are too general. Try providing specific significant conclusions and give recommendations for future work.

We agree with the reviewer’s suggestion and have attempted to expand and re-word the conclusions to highlight more specific conclusions of the study.

**Reviewer #3:**

The paper titled "Climate change will increase heat-related illness risk in Grand Canyon National Park visitors" is a very important and significant work that provides valuable insights into the potential impact of climate change on the health and well-being of Grand Canyon National park visitors. The study presents a clear and well-structured argument, supported by a thorough analysis of the data, and provides a strong foundation for future research in this area. One of the strengths of the paper is its descriptive nature, providing a detailed account of the study methods and results. The use of statistical models and the careful consideration of the limitations of the data and methods is commendable.

However, it would be beneficial for the manuscript to include more formal explanations of certain concepts, such as the standard heat index equation used in the study. Additionally, it will be more informative if the authors provide more details about the study area and its characteristics.

We thank reviewer 3 for their comments.

We chose not to include the equation in the text because 1) the method is well described by NOAA elsewhere, and 2) heat index, though tested in our analysis as a possible predictor, was not significant under forward selection and therefore not included in the final regression, so we felt that it added unnecessary technical detail to the manuscript. Therefore, we have simplified the text about heat index to make it less confusing:

“We calculated heat index using standard methods described in U.S. National Oceanographic and Atmospheric Administration (NOAA, 2014).”

Additionally, we have added Figure 1, a map depicting the study area.
